# Interactive effect of oral anti-hyperglycaemic or anti–hypertensive drugs on the inhibitory and bactericidal activity of first line anti-TB drugs against *M*. *tuberculosis*

**DOI:** 10.1371/journal.pone.0292397

**Published:** 2023-11-30

**Authors:** Priyanka Trivedi, Vinita Chaturvedi

**Affiliations:** Biochemistry and Structural Biology Division, Central Drug Research Institute, Lucknow, UP, India; University of Cape Town, SOUTH AFRICA

## Abstract

Co-existence of life style disorders, like, Diabetes or Hypertension, increases risk of, treatment failure, deaths and developing drug-resistant TB. Concomitant administration of drugs to treat dual/multi-morbidities may alter their effectiveness, in additive/synergistic or adverse/antagonistic manner. We evaluated interactive effect of 7 anti-hyperglycaemic (HG) and 6 anti-hypertensive (HT) drugs on the inhibitory (MICs) and bactericidal (% killing of intracellular bacilli) activities of anti-TB drugs, Isoniazid (INH), Rifampicin (RFM), Ethambutol (EMB) and Streptomycin (STR) against *M*. *tuberculosis*. Five anti-HG drugs, namely, Acarbose, Acetohexamide, Glyburide, Repaglinide and Sitagliptin imparted either ‘additive’ or ‘no effect’ on the activities (inhibition or % killing) of all the four anti-TB drugs, as evident by their lower FICs (Fractional Inhibitory concentrations) and higher bacterial killing in combination. Metformin and Rosiglitazone, however, exerted adverse effect on the Ethambutol (FICs >2.0). All the six anti-HT drugs, namely, Atenolol, Hydrochlorothiazide, Ramipril, Valsartan, Nifedipine and Verapamil exerted either ‘additive’/’synergistic’ or ‘no effect’ on the activities of anti-TB drugs. These findings may help clinicians to select safe and helpful anti-HG or anti-HT drugs for TB patients, if, suffering with diabetes or hypertension like co-morbidities and receiving DOTs (a set regimen for the treatment of TB based on the WHO guidelines).

## Introduction

Globally recent past (between 2000 and 2017) witnessed a 42% decline in deaths and 1.5% annual fall in the incidence rate of Tuberculosis (TB). In spite of these figures, TB continues to be a major health threat worldwide. Estimation of TB incidence during the COVID-19 pandemic is much more difficult than previously. In an estimated global total, 10.6 million people fell ill with TB in 2021 with an estimated 1.6 million deaths. This was up from best estimates of 1.5 million in 2020 and 1.4 million, deaths in 2019 [1]. The disease can affect anyone or everyone, but there are specific population groups who are at higher risk of acquiring TB infection and progressing to disease once infected. These risk factors can be divided into two groups, first socio-economic, e.g., people living in, Congregate settings (prisons), endemic areas having high risk of transmission of *M*. *tuberculosis*, Poverty (Malnutrition, Homelessness), Alcoholism, tobacco smoking and drug abuse, etc. The second group includes medical co-morbidities, i.e., HIV co-infection, non-communicable diseases (NCDs) [2] such as Diabetes mellitus (DM) [3, 4], cardiovascular diseases (CVDs) [5], lung cancer and chronic respiratory diseases [2, 5]. Reports indicate, that, risk of developing TB gets many folds higher with these risk factors, e.g. DM, Tobacco smoking and alcoholism, individually, increases risk by 2-3-fold, HIV-Co-infection increases the risk by 15-20-fold.

In the scenario of dual co-morbidity of TB and DM, one disease automatically clubbed in, is Hypertension. Because, occurrence of Hypertension is very common in diabetic patients and this combination contributes immensely to increase the risk of cardiovascular disease. According to the IDF Diabetes Atlas 2021 reports 10.5% of the adult population of age range 20–79 years has diabetes and by the year 2045, approximately 783 million, will be living with diabetes with an increase of 46% [6]. According to WHO report (2023), worldwide, about 422 million people have diabetes and 1.5 million deaths directly attributed to diabetes each year [7]. LMICs (Low- and Middle- Income Countries) are particularly expected to suffer with highest increases mainly in type 2 diabetes [2]

Worldwide, many studies showed concomitant diabetes mellitus in about 5–30% of TB patients [8–11]. The relative risk for TB among diabetic patients has been reported is 3.11-fold higher for Drug susceptible TB [12], whereas, 2.1-fold higher in multidrug-resistant (MDR) TB [13]. Reports showed a bidirectional relationship between TB and diabetes, and both affects the presentation of each other, negatively [14]. On one side, diabetes increases the risk for developing more severe forms of TB and also affects its presentation, e.g., probability of reactivation has been found higher in patients with DM (as compared to non-diabetics), as evident by the relatively higher involvement of lower lobes of Lungs in the former [15]. Further, rates of haemoptysis, fever, and atypical presentations are also found higher in patients with dual morbidity as compared to non-diabetics with TB [16, 17]. Diabetes is associated with increased risk of, developing active TB, deaths during TB treatment, failure or longevity of treatment and relapse after treatment completion. On the other side, TB may worsen glycaemic control or lead to Impaired Glucose Tolerance (IGT) among TB patients [18]. Commonly, IGT normalizes after the cure of TB by treatment, but chances of developing type 2 diabetes in the future get increased [15]. Also, TB infection may adversely affect functioning of Pancreas that may subsequently cause IGT and TB pancreatitis [19].

Among other commonly occurring life style NCDs, Cardiovascular diseases (CVDs) are responsible for one third of deaths and of which more than 75% occur in LMICs [20]. According to an estimate of WHO report 2023, Worldwide, 1.28 billion adults aged 30–79 years have hypertension, most (two-thirds) living in low- and middle-income countries. One of the global targets for non-communicable diseases is to reduce the prevalence of hypertension by 33% by 2030 [21]. Hypertension plays key role in the development of CVDs and is also found responsible for a large number of deaths due to heart and kidney disease [5]. In addition to lifestyle factors, chronic infections, like TB, are also reported to increase the risk of hypertension and CVDs [22]. Nevertheless, a reverse association may also possibly exist, that hypertension may lead to an increased risk of developing TB. The reported prevalence of hypertension in TB patients varies from 0% to 50% [20, 21]. In this regard, it is suggested that, triggering of immunological responses due to TB infection can impair endothelial function and may lead to an increased risk of CVDs and possibly hypertension [5, 23]. Available data indicate that TB may play a role in the pathogenesis of CVDs through molecular mimicry and auto-immunity mediated by anti-HSP65 antibodies [23]. Parenchymal destruction of the lung tissues due to TB [24] may affect the vascular structure and cause vasculitis and endarteritis, subsequently leading to a reduced cross-sectional area of the pulmonary vasculature and thereby pulmonary hypertension. Additionally, TB infection in kidneys may also cause hypertension, by, parenchymal destruction of the renal tissues and impairment of renal functions including ability of the kidney to regulate blood pressure [25, 26]. Vice-versa, hypertension may exert subtle effects on the immune system [27], which can increase the risk of TB.

The above mentions together strongly point out the severity of problem of multi-morbidity. In this multi-morbidity situation, one disease adversely affects presentation of other disease(s). Moreover, concomitant administration of drugs used to treat such diseases may exert effect(s) on the, therapeutic potential, toxicity and pharmacological activity (ADME) of each other. Therapeutic efficacies may get altered in synergistic, additive or antagonistic manner. In the present study, we aimed to determine the interactive effect of selected oral, anti-hyperglycaemic (HG) and anti-hypertensive (HT) drugs, belonging to different chemical classes, on the inhibitory and bactericidal activities of the first line anti-TB drugs, namely, Isoniazid (INH), Rifampicin (RFM), Ethambutol (EMB) and Streptomycin (STR), against extracellular and intracellular *M*. *tuberculosis*. Rationale behind studying these interactive effects is the long durations of treatments of Tuberculosis as well as the two co-morbidities, i.e., Diabetes and Hypertension.

Results of the study suggest, that, except, Metformin and Rosiglitazone, other anti-HG or anti-HT drugs studied may be given safely to TB patients receiving DOTs and suffering with either of the two co-morbidities.

## Materials and methods

### Materials

#### Mycobacteria

*Mycobacterium tuberculosis* H37Ra (*M tb*, ATCC 25177, USA) was grown and maintained on Lowenstein-Jensen (L-J) medium. The bacterial suspension of three-week-old culture (on L-J medium) prepared in Middle brook 7H9 broth (Difco^TM^, MB from BD, Becton Dickinson, USA) containing 15% glycerol (as cryopreservative) was stored in aliquots at -80°C. Viable bacterial counts (colony forming units, cfu) were determined by plating the bacteria (50 μl, diluted in MB broth) on MB 7H11 agar medium supplemented with 10% OADC and 0.5% glycerol and incubating (37°C) until countable colonies appeared (3–4 weeks).

#### Drugs

All the drugs, namely, anti-tubercular drugs- Isoniazid (INH), Rifampicin(RFM), Ethambutol (EMB) and Streptomycin(STR), Oral anti-hyperglycaemic drugs (HG)- Acarbose (ACB), Acetohexamide (ACT), Glyburide (GLY), Metformin (MET), Repaglinide (REPA), Rosiglitazone (ROSI) and Sitagliptin (SITA) and Oral anti-hypertensive drugs (HT) ‐ Atenolol (ATE), Hydrochlorothiazide (HCTZ), Prazosin (PRA), Ramipril (RAM), Valsartan (VAL), Nifedipine(NIF) and Verapamil (VER) were purchased from Sigma-Aldrich. All other chemicals used in this study were also purchased from Sigma-Aldrich, otherwise mentioned.

General information towards the functioning of anti-HG and anti-HT drugs is summarised in **Table 1**.

**Table 1:** General information about the functioning of anti-hyperglycaemic (HG) and anti-hypertensive (HT) drugs.

S. No.	Anti-hyperglycaemic (HG) Drugs		
	Name	Generic name	Functioning
1.	Acarbose (ACB)	Acarbose	Delays digestion of carbohydrates, slows glucose absorption, thereby reduces postprandial glucose blood concentrations.
2.	Acetohexamide (ACT)	Acetohexamide	Lowers blood sugar by stimulating pancreatic beta cells to secrete insulin and helps the body to utilise insulin efficiently.
3.	Glyburide (GLY)	Glibenclamide	Increases insulin secretion from beta cells in the pancreas.
4.	Metformin (MET)	Metformin hydrochloride	Decreases, production of glucose by liver, absorption of glucose by intestine, and improves insulin sensitivity by increasing uptake and utilization of peripheral glucose.
5.	Repaglinide (REPA)	Repaglinide	Stimulates insulin release from pancreatic beta cells.
6.	Rosiglitazone (ROSI)	Rosiglitazone maleate	Improves glycaemic control by improving insulin sensitivity. It mainly increases glucose uptake in skeletal muscles.
7.	Sitagliptin (SITA)	Sitagliptin Phosphate	Increases levels of natural substances called incretins, which in turn, increase insulin release (especially after a meal), improves glucose tolerance and decreases the production of glucose by liver.
**Anti- hypertensive (HT) drugs**			
1.	Atenolol (ATE)	Atenolol	Blocks the effects of the hormone epinephrine (adrenaline) thereby slows down and smoothen beating of heart. It also widens veins and arteries which lowers blood pressure and improves blood flow.
2.	Hydrochlorothiazide (HCTZ)	Hydrochlorothiazide	Increases excretion of sodium and chloride in the urine by affecting the electrolyte reabsorption at distal renal tubule.
3.	Prazosin (PRA)	Prazosin	Decreases blood pressure by dilating blood vessels.
4.	Ramipril (RAM)	Ramipril	Reduces reabsorption of sodium and water from the kidneys, relaxes smooth muscles in the arterioles thereby lowers blood pressure.
5.	Valsartan (VAL)	Valsartan	Relaxes and widens blood vessels thereby lowers blood pressure.
6.	Nifedipine (NIF)	Nifedipine (Procardia XL)	Inhibits the entry of calcium ions in vascular smooth muscle and myocardial cells, that reduces blood pressure and increases oxygen supply to the heart.
7.	Verapamil (VER)	Verapamil	Relaxes blood vessels and increases the supply of blood and oxygen to the heart by affecting movement of calcium into the cells of the blood vessels and heart. Also, it slows electrical activity in the heart to, control the rate of heart beat and reduces the work load of heart.

## Methods

### Determination of inhibitory activity against *M*. *tuberculosis H37Ra*

This *in vitro* assay evaluates potential of drugs/molecules to inhibit the growth of extracellular *Mtb*. Inhibitory potential was determined as Minimum Inhibitory Concentrations (MICs) of all the drugs, namely, anti-tubercular (anti-TB), anti-hyperglycaemic (HG) and anti-hypertensive (HT), against *Mtb* H*37*Ra by Agar Proportion assay [28]. Briefly, stocks (5.0 mg/ml) of all the drugs were prepared in Dimethyl sulphoxide (DMSO). Serial 2.0-fold dilutions from stocks were also made in DMSO. To 1.9 mL MB 7H11 agar medium (in tubes, temp. 45–50 ºC, with OADC supplement), 0.1 mL of drug(s) dilution was added. The contents were mixed and allowed to solidify as slants. From the cryopreserved stocks of *M tb*, bacterial suspension (1 mg/ml equivalent to 10^8^ bacilli approximately) was made in normal saline containing 0.05% Tween-80. Ten μl of 1:10 dilution of this suspension (∼10^5^ bacilli) is inoculated into each tube and incubated at 37 ºC for 4 weeks. The lowest concentration of a drug up to which there was no visible growth of bacilli was its Minimum Inhibitory Concentration (MIC). Anti-TB drugs, also served as positive control and the DMSO (only) served as negative control.

### Cytotoxicity of drugs towards Mouse Bone marrow derived Macrophages (MBMDM_Φ_)

All the Drugs were tested for cytotoxicity towards Primary macrophages obtained from the bone marrow of Swiss mouse, using MTT assay [29]. [Please note: Prior approval of the *in vivo* experiments in mouse, from the Institute’s ethics committee (IAEC approval for small animal experimentation No. IAEC/2012/15/Renew 8/dated 14.6.2019, for one year) has been taken]. Mouse was euthanized by exposure to CO_2_ and the femur bones were dissected out. The bones were trimmed at each end, and the marrow was flushed out (using 26-gauge needle) with 5 mL of Dulbecco’s minimal essential medium (DMEM) supplemented with 10% FBS (Gibco, USA), 15% L-929 fibroblast (Obtained from Institutes Cell Culture Facility) conditioned supernatant and non-essential amino acids (1.0% final concentration). Cells were washed twice and plated in 96 well tissue culture plates at a concentration of 10^5^ cells per well (/100 μL) in supplemented DMEM. For adherence monolayers were then incubated 5 days with change of medium after 3 days at 37°C in 5% CO_2_. Different concentrations of test drugs/ DMSO (in DMEM containing 10% FBS only) were added 12 h after the adherence of cells. After 48 h, 20 μL of MTS solution (Promega, USA) was added to each well and incubated for 2 h at 37°C in 5% CO_2_. Absorbance was read at 490 nm using a plate reader. Absorbance shown by DMSO containing wells was taken as 100% survivors. A drug was considered toxic if it causes 50% inhibition in cell growth at a concentration 10-fold higher than its MIC.

### Interactive effect of anti-hyperglycaemic (HG) or anti-hypertensive (HT) drugs on the activity of anti-TB drugs

#### Effect on the inhibitory activity against extracellular *M*. *tuberculosis*

Interaction between anti-TB drugs, and anti-hyperglycaemic (HG) drugs or anti-hypertensive (HT) drugs was assessed by determining the Fractional Inhibitory Concentrations (FICs) [30–33] of anti-TB drugs, INH, RFM, EMB and STR, against *Mtb*, in presence of anti-HG or anti-HT drugs. For FIC determination, test concentrations of anti-TB drugs used were, two-fold dilutions starting from 2.0 x MIC, i.e. 1.0 x, 0.5 x, 0.25 x, 0.125 x and 0.0625 x MIC. Each concentration of anti-TB drug was tested in combination with the single test concentration, i.e., 0.50 x MICs of anti-HG or anti-HT drugs (at which the individual drug did not show any bacterial inhibition). Fifty μl each of the two drugs (containing the required final concentration) were added to the 7H11 medium. Remaining procedure was same as that used for MIC determination [28].

Fractional Inhibitory Concentrations (FICs) were calculated by dividing the MIC of drug in combination by the MIC of that drug alone (FIC = MIC in combination / MIC alone) [30]. FICs, < 1.0 indicate ‘additive’ effect, > 1.0 indicate ‘adverse’ effect and FIC = 1.0 indicate no interactive effect (‘No effect’) of anti-HG or anti-HT drugs on the inhibitory activity of anti-TB drugs against *M*. *tuberculosis*. The term ‘No effect’ indicate that two drugs act independently of each other.

#### Effect on the bactericidal activity against intracellular *M*. *tuberculosis*

Determination of the killing of intracellular *Mtb* was done by MBMDM_Φ_ model. This model mimics growth environment of natural infection and demonstrate the ability of a drug or molecule to penetrate host cell membrane and phagocytic vacuole, bacilli residing within the vacuole and reach to the desired drug target of the bacilli. In addition, it also serves as the hypoxia induced model of latent TB infection, because tissue concentration of oxygen is considerably lower than that in the ambient air.

The study protocol was approved by the Institute’s (CDRI’s) Animal Ethics Committee, No. IAEC/2012/15/Renew8/ dated 14.6.2019 for a year. Mouse bone marrow-derived macrophages (BMDM) were prepared from femurs and tibia of Swiss mice. Cells were harvested in DMEM medium by flushing out the bone marrow using a syringe. Cells were collected by centrifugation (1000g x 15 min) and re-suspended in ‘complete medium’ (DMEM with 10% foetal bovine serum (FBS), 15% culture supernatant of L-929 cells and 1% non-essential amino acids). After counting, 10^6^/ ml cells were dispensed in 48- well culture plates, cells 350 μl/well were incubated for 5 days in a CO2 incubator (37°C, 5% CO2) for adherence and maturation.

Matured BMDM cells were infected with *Mtb* diluted in complete medium without antibiotics. Aggregated bacilli were dispersed by a short pulse (10s) of sonication which did not affect their viability (ascertained by cfu counts). Cells were infected with *Mtb* using a multiplicity of infection (MOI) of 5 (i.e., bacilli: macrophage, 5:1) e.g., 5x10^6^ bacilli/well/350μl, and incubated for 5 days (37°C, 5% CO_2_). Later, the macrophage monolayer was washed extensively with DMEM to remove extracellular bacilli.

Dilutions of anti-TB (to prepare i.e., 4.0 x, 2.0 x, 1.0 x, 0.50 x or 0.25 x, MICs), anti-HG and anti-HT drugs were prepared in complete medium. Drug dilutions or drug combinations or medium alone (as a positive control) were dispensed in duplicate wells (350 *μ*l/ well). For drug combinations, respective MICs of anti-TB drug(s) and test concentrations of anti-HG or anti-HT drugs were made in such a way that the required concentration is achieved in 175 *μ*l, so that final volume of two drugs will be 350 *μ*l. Plates were incubated for further 5 days (37°C, 5% CO_2_). For the determination of intracellular cfu, cells were lysed with saponin (0.1%, 100 *μ*l/well, 15 min) and the lysates, after diluting in complete medium, were plated on MB 7H11 agar and incubated for 3–4 weeks (37°C, 5% CO_2_).

Killing of intracellular *Mtb* was assessed by counting the viable bacilli (colony forming units or CFUs) in lysates of the drug-treated or untreated infected macrophages. Percent (%) bacterial killing was calculated by considering CFUs of untreated wells (only medium) as 100% bacteria.

## Results

Minimum inhibitory concentrations (MICs) against *M tuberculosis* H37Ra and CC50 values (Cytotoxic Concentration that causes 50% reduction in cell viability) of all the drugs are shown in **Table 2**. Importantly, all drugs (anti-TB, anti-HG and anti-HT), except anti-HT drug Prazosin, were found non-cytotoxic towards Mouse Bone marrow derived macrophages (MBMDM_Φ_). Therefore, drug interaction experiments were not performed with Prazosin.

**Table 2:** Inhibitory activity (MICs) against *M tuberculosis* and Cytotoxicity (CC50) towards mouse macrophages, of anti-tubercular (TB), anti-hyperglycaemic (HG) and anti-hypertensive (HT) drugs.

S. No.	Anti-tubercular (TB) drugs (Drug class)	MIC (μg/ml)	CC50 (μg/ml)
1	Isoniazid (hydrazide of isonicotinic acid)	0.10	>100.0
2	Rifampicin (derivative of rifamycin SV)	0.40	>100.0
3	Ethambutol (derivative of ethylenediamine)	4.00	>100.0
4	Streptomycin (aminoglycoside antibiotic from *S griseus*)	2.00	>100.0
**Anti-hyperglycaemic (HG) drugs** (Drug class)			
1	Acarbose (ACB) (Alpha-glucosidase inhibitor)	>100.0	>250.0
2	Acetohexamide (ACT) (1^st^ generation-Sulfonylureas)	>100.0	>250.0
3	Glyburide (GLY) (2^nd^ generation-Sulfonylureas)	>100.0	>250.0
4	Metformin (MET) (Biguanide)	50.0	>250.0
5	Repaglinide (REPA) (Thiazolidinediones)	>100.0	>250.0
6	Rosiglitazone (ROSI) (Meglitinide)	50.0	>250.0
7	Sitagliptin (SITA) (Dipeptidyl peptidase IV inhibitors)	>100.0	>250.0
**Anti- hypertensive (HT) drugs** (Drug class)			
1	Atenolol (ATE) (Beta-blockers)	>100.0	>250.0
2	Hydrochlorothiazide (HCTZ) (Thiazide diuretics)	>100.0	>250.0
3	Prazosin (PRA) (Alpha-1 Blocker)	>100.0	25.00
4	Ramipril (RAM) (#ACEIs)	>100.0	>250.0
5	Valsartan (VAL) (Angiotensin II antagonists)	50.0	>250.0
6	Nifedipine (NIF) (CCB*s- dihydropyridines)	50.0	>250.0
7	Verapamil (VER) (CCBs- non-dihydropyridines)	50.0	>250.0

Notes: 1. Cytotoxicity shown as CC_50,_ was determined towards Mouse bone marrow derived macrophages.

2. #ACEIs- Angiotensin Converting Enzyme Inhibitors.

3. *CCBs- Calcium channel blockers.

4. Values shown are average of two consecutive experiments.

### Interactive effect of anti-hyperglycaemic (HG) or anti-hypertensive (HT) drugs on the activity of anti-TB drugs against *M tuberculosis*

#### Effect on the inhibitory activity of anti-TB drugs against extracellular *M*. *tuberculosis*

**Fig 1:**
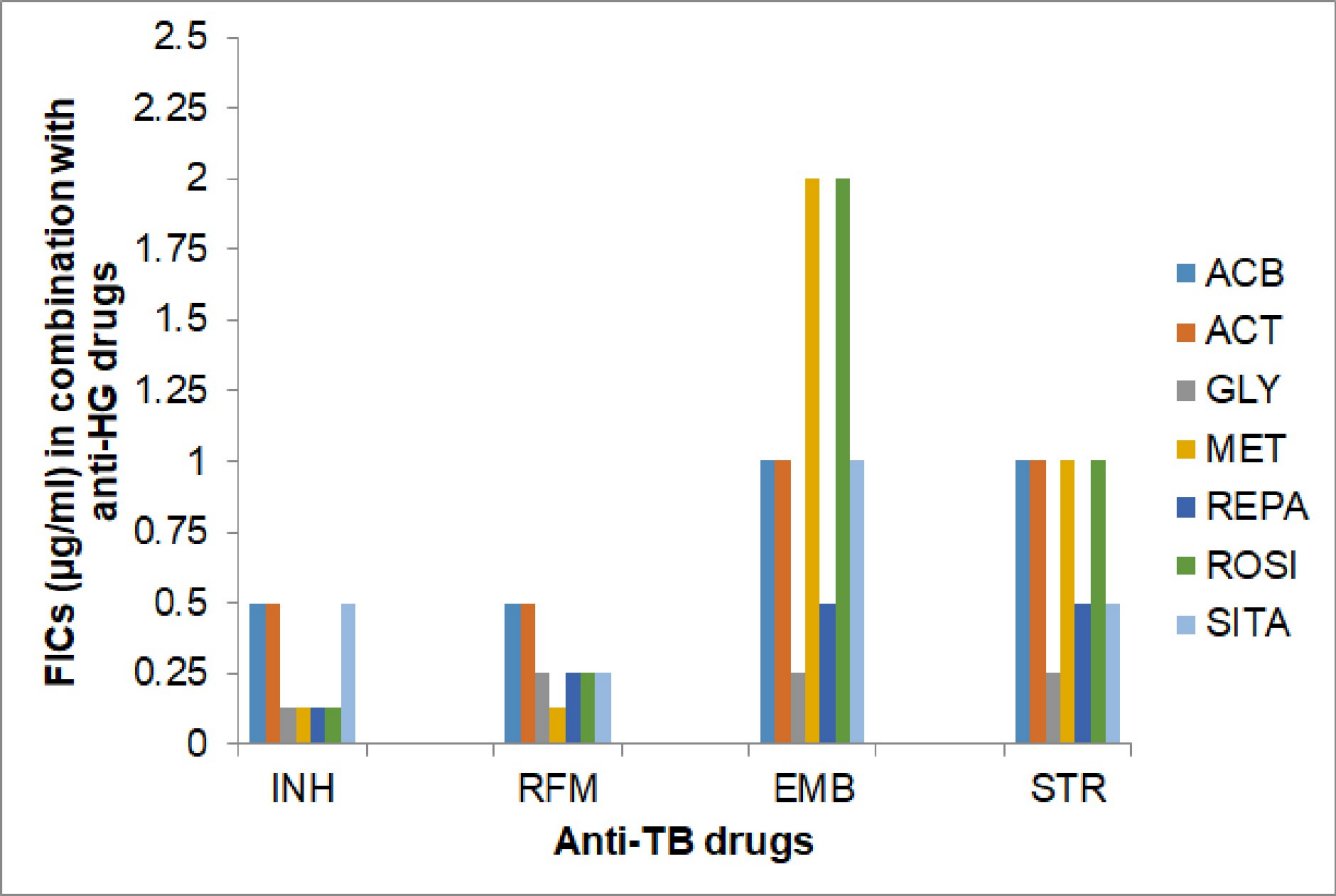
Interactive effect of anti-hyperglycaemic (HG) drugs on the inhibitory activity (MIC) of anti-TB drugs against *M tuberculosis*, shown as Fractional Inhibitory Concentrations (FICs). Notes: 1. Anti-TB drugs, INH-Isoniazid, RFM-Rifampicin, EMB-Ethambutol and STR- Streptomycin, against *Mtb*. 2. Anti-hyperglycaemic Drugs: ACB- Acarbose, ACT-Acetohexamide, GLY- Glyburide, MET- Metformin, REPA- Repaglinide, ROSI- Rosiglitazone, SITA- Sitagliptin. 3. Data shown are average of two consecutive experiments.

**Fig 2:**
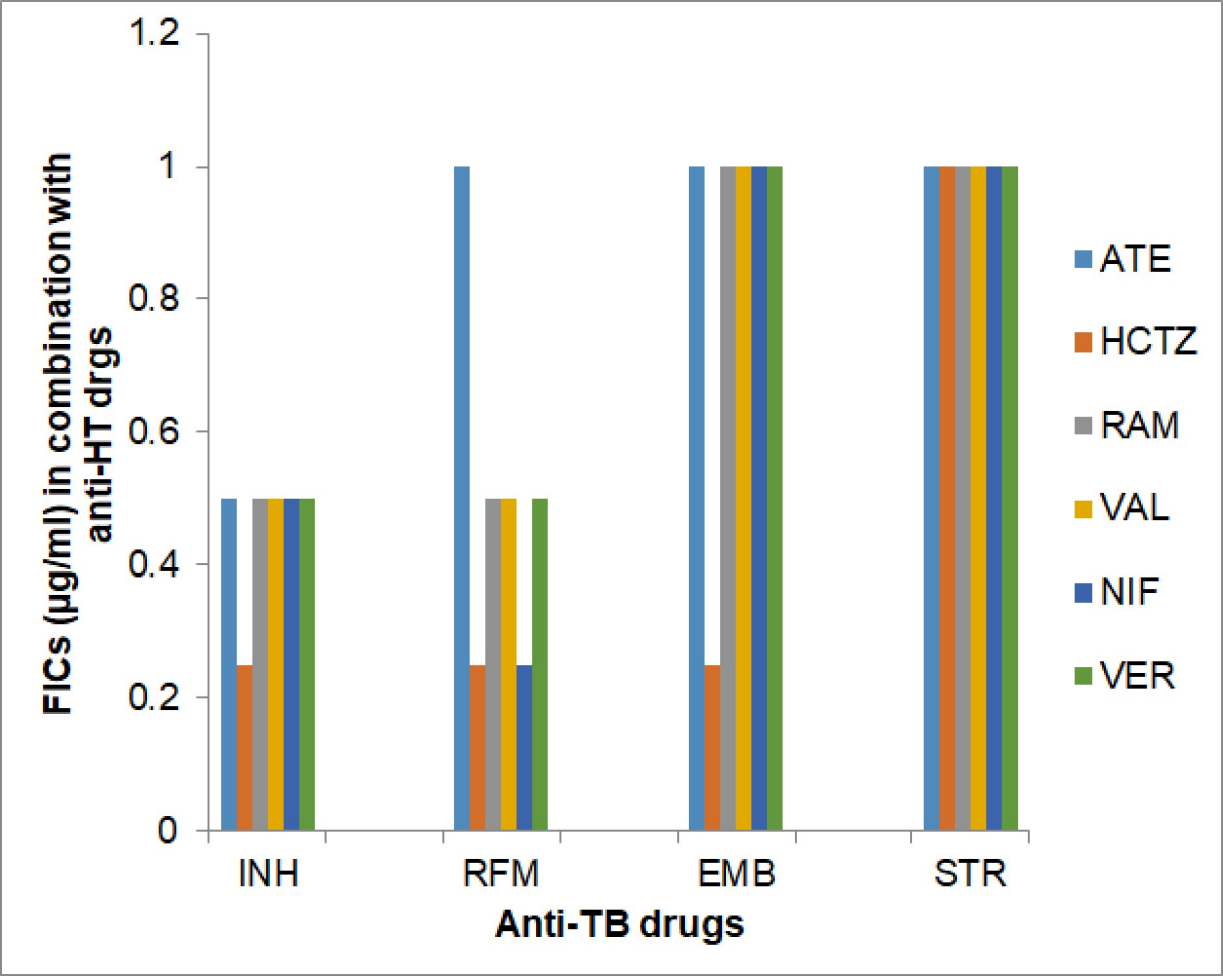
Interactive effect of anti-hypertensive (HT) drugs on the inhibitory activity (MIC) of anti-TB drugs against *M*. *tuberculosis*, shown as Fractional Inhibitory Concentrations (FICs). Anti-hypertensive (HT) Drugs: ATE-Atenolol, HCTZ- Hydrochlorothiazide, RAM-Ramipril, VAL-Valsartan, NIF- Nifedipine, VER-Verapamil. Notes: remaining details are same as in Fig 1.

Effect of anti-hyperglycaemic (anti-HG) and anti-hypertensive (anti-HT) drugs on the inhibitory activity (MICs) of the anti-TB drugs against *M*. *tuberculosis*, assessed as their Fractional Inhibitory Concentration (FICs) in combination, is shown, respectively, in **Table 3** and **Figs 1 and 2**.

**Table 3:** Summary of interactive effect of anti-hyperglycaemic (HG) and anti-hypertensive (HT) drugs on the inhibitory activity of anti-TB drugs against *M tuberculosis*.

Combination with, Anti- HG drugs	Inhibitory Concentrations (μg/ml) of anti-TB drugs									
	INH (MIC 0.10)		RFM (MIC 0.40)		EMB (MIC 4.0)			STR (MIC 2.0)		
	in combination		in combination		in combination			in combination		
	MIC & [Fold reduction in MIC]	FIC	MIC & [Fold reduction in MIC]	FIC	MIC & [Fold reduction in MIC]		FIC	MIC & [Fold reduction in MIC]		FIC
+ ACB	0.05 [2.00]	0.50 *Additive	0.20 [2.00]	0.50 Additive	4.00 [None]		1.00 No effect	2.00 [None]		1.0 No effect
+ACT	0.05 [2.00]	0.50 Additive	0.20 [2.00]	0.50 Additive	4.00 [None]		1.00 No effect	2.00 [None]		1.00 No effect
+GLY	0.0125 [8.00]	0.125 Additive	0.10 [4.00]	0.25 Additive	1.00 [4.00]		0.25 Additive	0.50 [4.00]		0.25 Additive
+ MET	0.0125 [8.00]	0.125 Additive	0.05 [8.00]	0.125 Additive	>8.00 [>2.0]		>2.00 Adverse	2.00 [None]		1.00 No effect
+REPA	0.0125 [8.00]	0.125 Additive	0.10 [4.00]	0.25 Additive	2.00 [2.00]		0.50 Additive	1.00 [2.00]		0.50 Additive
+ROSI	0.0125 [8.00]	0.125 Additive	0.10 [4.00]	0.25 Additive	>8.00 [>2.0]		>2.00 Adverse	1.00 [2.00]		0.50 Additive
+SITA	0.05 [2.00]	0.50 Additive	0.10 [4.00]	0.25 Additive	4.00 [None]		None No effect	1.00 [2.00]		0.50 Additive
With Anti-HT drugs										
+ ATE	0.05 [2.00]	0.50 Additive	0.40 [None]	1.00 No effect		4.00 [None}	1.00 No effect		2.00 [None]	1.00 No effect
+ HCTZ	0.025 [4.00]	0.25 Additive	0.10 [4.00]	0.25 Additive		1.00 [4.00]	0.25 Additive		2.00 [none]	1.00 No effect
+ RAM	0.05 [2.00]	0.50 Additive	0.20 [2.00]	0.50 Additive		4.00 [None]	1.00 No effect		2.00 [None]	1.00 No effect
+ VAL	0.05 [2.00]	0.50 Additive	0.20 [2.00]	0.50 Additive]		4.00 [None]	1.00 No effect		2.00 [None]	1.00 No effect
+ NIF	0.05 [2.00]	0.50 Additive	0.10 [4.00]	0.25 Additive]		4.00 [None]	1.00 No effect		2.00 [None]	1.00 No effect
+ VER	0.05 [2.00]	0.50 Additive	0.20 [2.00	0.50 Additive		4.00 [None]	1.00 No effect		2.00 [None]	1.00 No effect

Notes: 1. *Interactive effect was assessed by determining the Fractional Inhibitory Concentration(s) (FICs, MIC of drug in combination / MIC of drug alone) of anti-TB drugs, INH-Isoniazid, RFM-Rifampicin, EMB-Ethambutol and STR -Streptomycin, against Mtb, in combination with 0.5x MIC of anti-hyperglycaemic (HG) or anti-hypertensive (HT) drugs. Drugs which showed MIC >100.0 μg/ml were combined at 100.0 μg/ml.

2. Anti-hyperglycaemic (HG) drugs ‐ ACB- Acarbose, ACT-Acetohexamide, GLY- Glyburide, MET- Metformin, REPA- Repaglinide, ROSI- Rosiglitazone, SITA- Sitagliptin.

Anti-hypertensive (HT) drugs ‐ ATE- Atenolol, HCTZ- Hydrochlorothiazide, RAM- Ramipril, VAL- Valsartan, NIF- Nifedipine and VER- Verapamil.

3. Interactive effect(s) was decided on the value of FIC as follows

if MIC in combination is equal to MIC alone, FIC = 1.00; ‘No effect’.

if MIC in combination is lesser than MIC alone, FIC <1.00; ‘Additive’ effect.

if MIC in combination is higher than MIC alone, FIC >1.00; ‘Adverse’ effect.

4. Data shown are average of two consecutive experiments.

#### Effect of anti- hyperglycaemic (HG) drugs

Among the seven anti-HG drugs tested, two drugs, namely, Glyburide and Repaglinide showed ‘additive’ effect (FIC < MIC) on all the four anti-TB drugs. Both drugs, Glyburide and Repaglinide, showed highest ‘additive’ effect on the activity of INH, as indicated by maximum, 8.0-fold reduction in the MIC of INH, i.e., FIC 0.125 μg/ml. Anti-HG drug Sitagliptin exerted ‘additive’ effect on INH, RFM and STR and ‘No effect’ (FIC = MIC, i.e., FIC = 1) on EMB [**Table 3**]. Anti-HG drugs, Acarbose and Acetohexamide exerted, additive effect on INH and RFM and ‘No effect’ on the inhibitory activity of EMB and STR. Anti-HG drug, Rosiglitazone showed adverse effect on Ethambutol (FIC >2.0) however, the drug exerted ‘additive’ effect on other three anti-TB drugs. Similarly, the anti-HG drug Metformin also imposed adverse effect on the inhibitory activity of Ethambutol. Although, Metformin exerted substantial ‘additive’ effect on the inhibitory activity of both INH and RFM, i.e., FIC 0.125 μg/ml and ‘no effect’ on the activity of STR [**Table 3 and Fig 1**]. These observations suggest that because Metformin and Rosiglitazone exerted an adverse effect on the anti-tubercular activity of EMB and that, these drugs, if given in combination(s) will impose negative and harmful impact on the TB treatment, hence should be avoided in TB patients receiving DOTs because Ethambutol play key role in the initial phase (Intensive phase) of treatment.

#### Effect of anti- hypertensive (HT) drugs

Among the six tested drugs, Hydrochlorothiazide (HCTZ) was found most suitable drug as it showed maximum ‘additive’ effect on 3 prime anti-TB drugs, namely, INH, RFM and EMB, by reducing MICs of these drugs by 4.0-fold [**Table 3 and Fig 2**]. Four anti-HT drugs, e.g., Ramipril, Valsartan, Nifedipine and Verapamil showed similar supportive (additive) response with two most important anti-TB drugs, INH and RFM and not effected inhibitory activity of other two anti-TB drugs EMB and STR. Atenolol showed ‘additive’ effect with only INH and imposed ‘no effect’ on the activity of other three anti-TB drugs. Some drug combinations provided higher ‘additive’ (nearly synergistic) effect, as evident by minimum FIC, i.e., 0.25μg/ml of anti-TB drugs, e.g., Combination of, Hydrochlorothiazide with, INH, RFM and EMB and Nifedipine with RFM. Importantly, all the anti-HT drugs produced either ‘additive’ or ‘No effect’ on the inhibitory activity of anti-TB drugs.

### Effect on the bactericidal activity of anti-TB drugs against intracellular *M*. *tuberculosis*

In a follow up, drugs’ combinations of anti-HG or anti-HT, which showed beneficial (additive) effect on the inhibitory activity (MICs) of anti-TB drugs, were also examined for their effect on the bactericidal activity of anti-TB drugs against intracellular *Mtb*. Experiments were performed with the *Mtb* multiplying within bone marrow derived macrophages of mouse.

**Fig 3:**
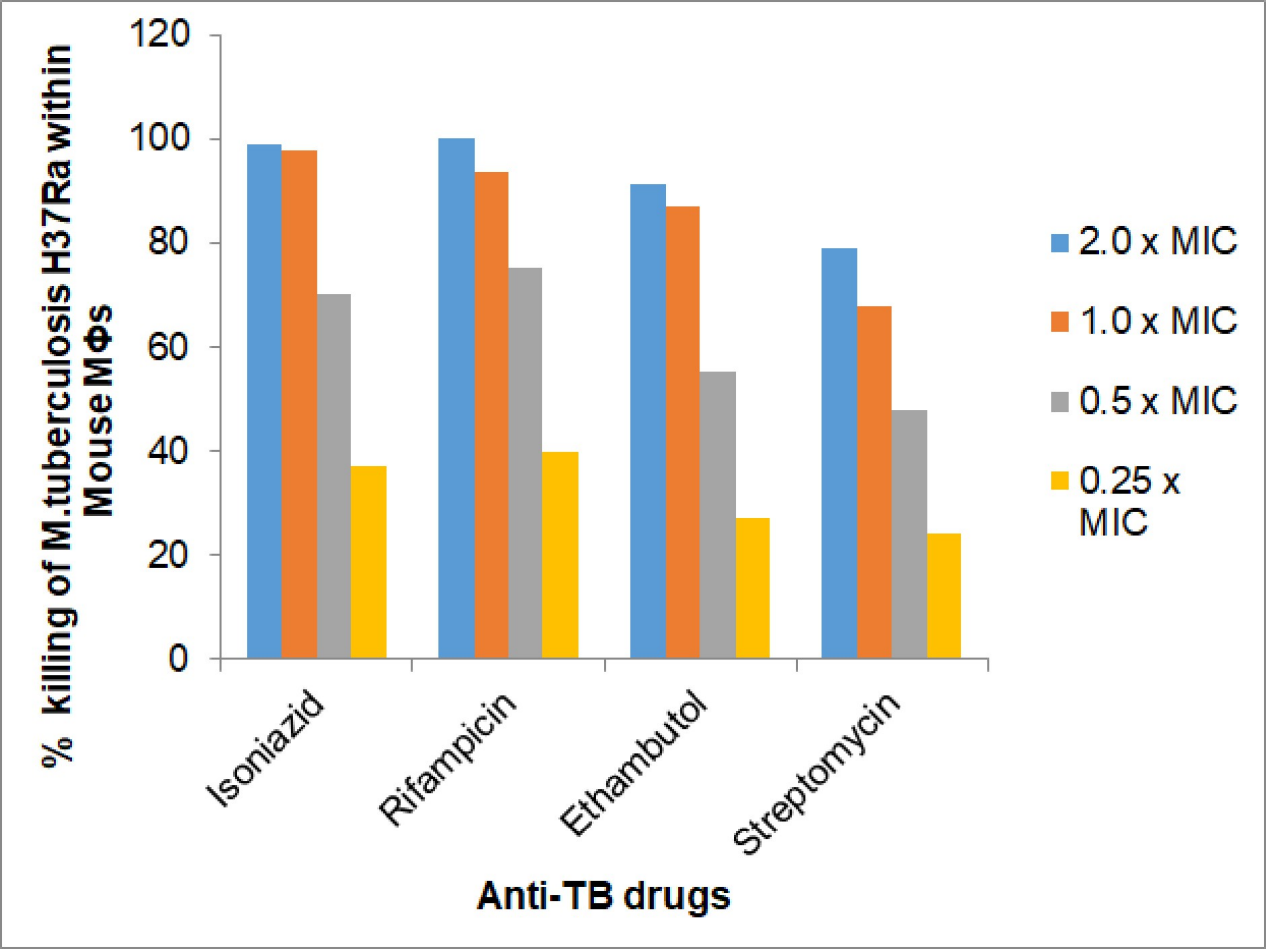
Killing of intracellular *M*. *tuberculosis* multiplying within mouse bone marrow derived macrophages by anti-TB drugs at different concentrations, e.g., 2.0 x, 1.0 x, 0.5 x and 0.25 x, MICs. Notes: Remaining details are same as in Fig 1.

In *ex vivo* experiments, concentration of a drug that is available in the intracellular environment plays important role in its activity. Availability of a drug in turn depends on its capacity to penetrate inside the cell. Therefore, in these experiments, concentrations equal to MICs of anti-HG or anti-HT drugs were used for combinations. Drugs which did not show inhibitory activity up to 100.0 μg/ml (MICs >100.0) were used at 100.0 ug/ml concentration. Bactericidal activity of anti-TB drugs was observed at their 0.25 x MIC, 0.50 x MIC, 1.0 x MIC and 2.0 x MICs [**Fig 3**]. In combination studies, concentrations of anti-TB drugs used were those which showed ‘additive’ activity with anti-HG or anti-HT drugs, *in vitro* studies.

#### Effect of anti- hyperglycaemic (HG) drugs

**Fig 4:**
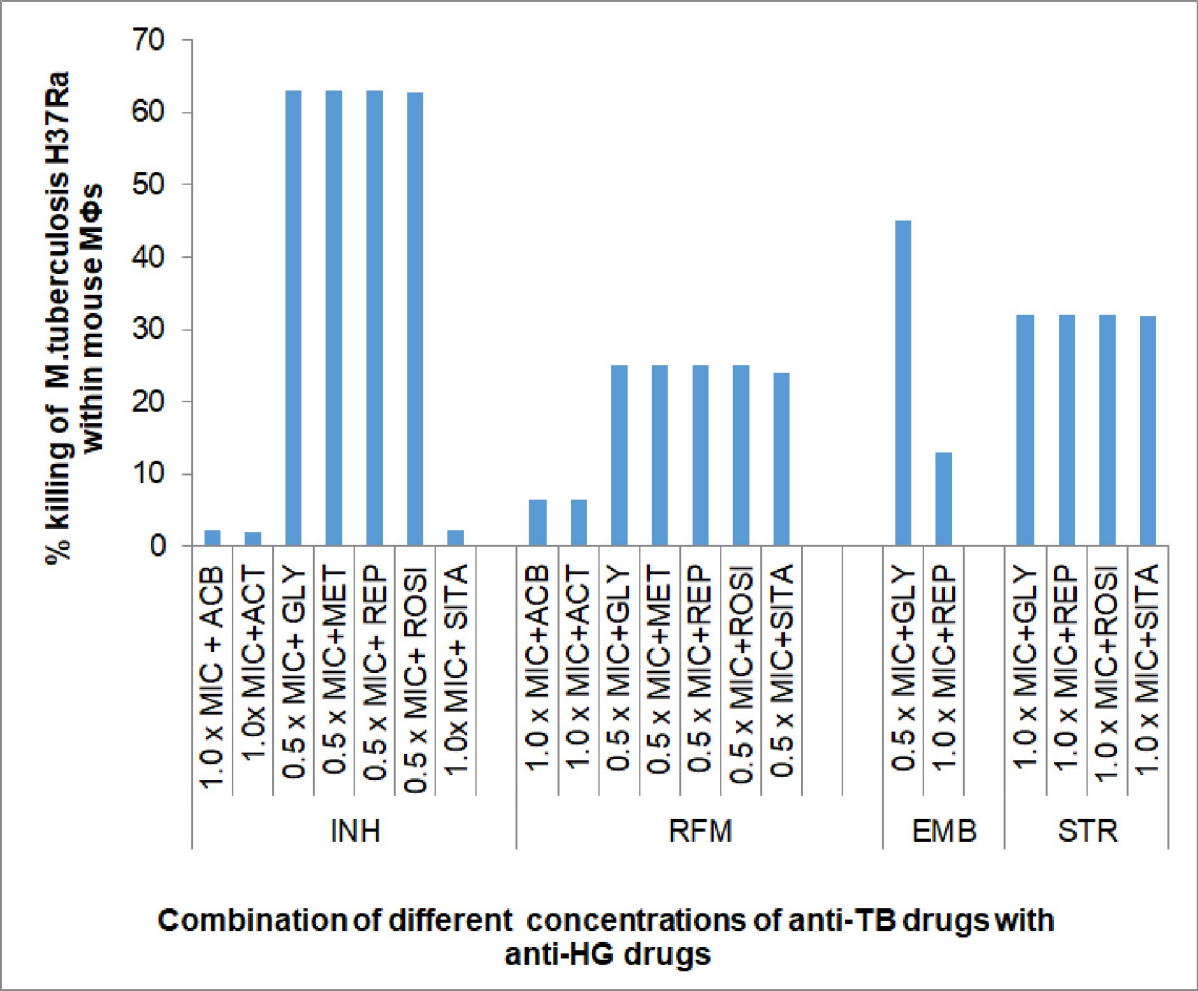
Interactive effect of anti-hyperglycaemic (HG) drugs on the bactericidal activity (% bacterial killing) of anti-TB drugs against intracellular *M*. *tuberculosis* multiplying within mouse bone marrow derived macrophages. Notes: Results are shown as per cent (%) bacterial killing by anti-TB drugs when used in combination with anti-HG drugs: ACB- Acarbose, ACT-Acetohexamide, GLY- Glyburide, MET- Metformin, REPA- Repaglinide, ROSI- Rosiglitazone, SITA- Sitagliptin. Notes: Remaining details are same as in Fig 1.

Results of interactive effect of anti-HG drugs on the bactericidal activity of anti-TB drugs [**Table 4 and Fig 4**] revealed, that, anti-HG drug, Glyburide showed ‘synergistic’ effect on the all the four anti-TB drugs as evident by increased bacterial killing by, INH (62.97% higher), RFM (25.0% higher), EMB (44.98% higher) and STR (32.0% higher) than the respective drugs alone. Anti-HG drugs, Repaglinide and Rosiglitazone imposed ‘synergistic’ effect on the bactericidal activity of three anti-TB drugs, INH, RFM and STR, as evident by a substantial increase of 25.0% to 63.0% in bacterial killing. Anti-HG drug, Sitagliptin brought an impressive increase in the bactericidal efficacy of INH (62.73% higher killing) and STR (31.73% higher killing). Anti-HG drugs, Acarbose and Acetohexamide were tested only in combination with INH or RFM, where they exerted either only marginal additive effect on RFM (i.e., about 6.50% higher bacterial killing) or ‘no effect’ on the bactericidal efficacy of INH. Results showed that, among the combinations tested no anti-hyperglycaemic (anti-HG) drug exerted any adverse effect on the bactericidal activity of anti-TB drugs.

**Table 4:** Summary of interactive effect of anti-hyperglycaemic (HG) and anti-hypertensive (HT) drugs on the bactericidal efficacy of anti-TB drugs against intracellular *M tuberculosis*.

Combination with, Anti- HG drugs	Bacterial killing by anti-TB drugs							
	INH		RFM		EMB		STR	
	in combination		in combination		in combination		in combination	
	% increase in Bacterial killing	Interactive effect	% increase in Bacterial killing	Interactive effect	% increase in Bacterial killing	Interactive effect	% increase in Bacterial killing	Interactive effect
+ ACB	2.17	None	6.52	M. Additive	Not done		Not done	
+ACT	2.06	None	6.41	M. Additive	Not done		Not done	
+GLY	62.97	Synergistic	25.00	Synergistic	44.98	Synergistic	32.00	Synergistic
+ MET	63.00	Synergistic	25.00	Synergistic	Not done		Not done	
+REPA	63.00	Synergistic	25.00	Synergistic	13.04	Additive	32.00	
+ROSI	62.73	Synergistic	25.00	Synergistic	Not done		32.00	
+SITA	2.16	None	23.91	Synergistic	Not done		31.73	
Anti-HT drugs								
+ ATE	1.09	None	Not done		Not done		Not done	
+ HCTZ	2.06	None	6.52	M. Additive	44.89	Synergistic	Not done	
+ RAM	1.06	None	None	None	Not done		Not done	
+ VAL	0.87	None	None	None	Not done		Not done	
+ NIF	2.17	None	6.41	M. Additive	Not done		Not done	
+ VER	1.09	None	6.20	M. Additive	Not done		Not done	

Notes: 1. Increase in bacterial killing was calculated by subtracting % bacterial killing by anti-TB drug alone at that concentration (x MIC) from % bacterial killing by the drug in combination (at the same respective x MIC used alone) with anti-HG or anti-HT drugs.

2. Concentrations of anti-TB drugs used in combination were selected on the basis of their inhibitory activity results.

3. Concentration(s) of anti-HG/anti-HT drugs used in combination were 1.0 x MIC. Drugs which showed MIC >100.0 μg/ml were combined at 100.0 μg/ml.

4. Not done- Based on the results of bacterial inhibition (*in vitro* MIC/FIC), ex vivo experiment was not performed.

5. Criteria for deciding Interactive effect of anti-HG/ anti-HT drugs on the bactericidal efficacy of anti-TB drugs was as follows

(a) ≤ 5% increase in the bacterial killing- showed as `None’.

(b) 6–10% increase in the bacterial killing- as, `Marginally additive (M. Additive)’.

(c) 11–20% increase in bacterial killing- as, `Additive’, and

(d) >20% increase in bacterial killing- as, ‘Synergistic’, effect.

6. Data shown are average of two consecutive experiments.

#### Effect of anti- hypertensive (HT) drugs

**Fig 5:**
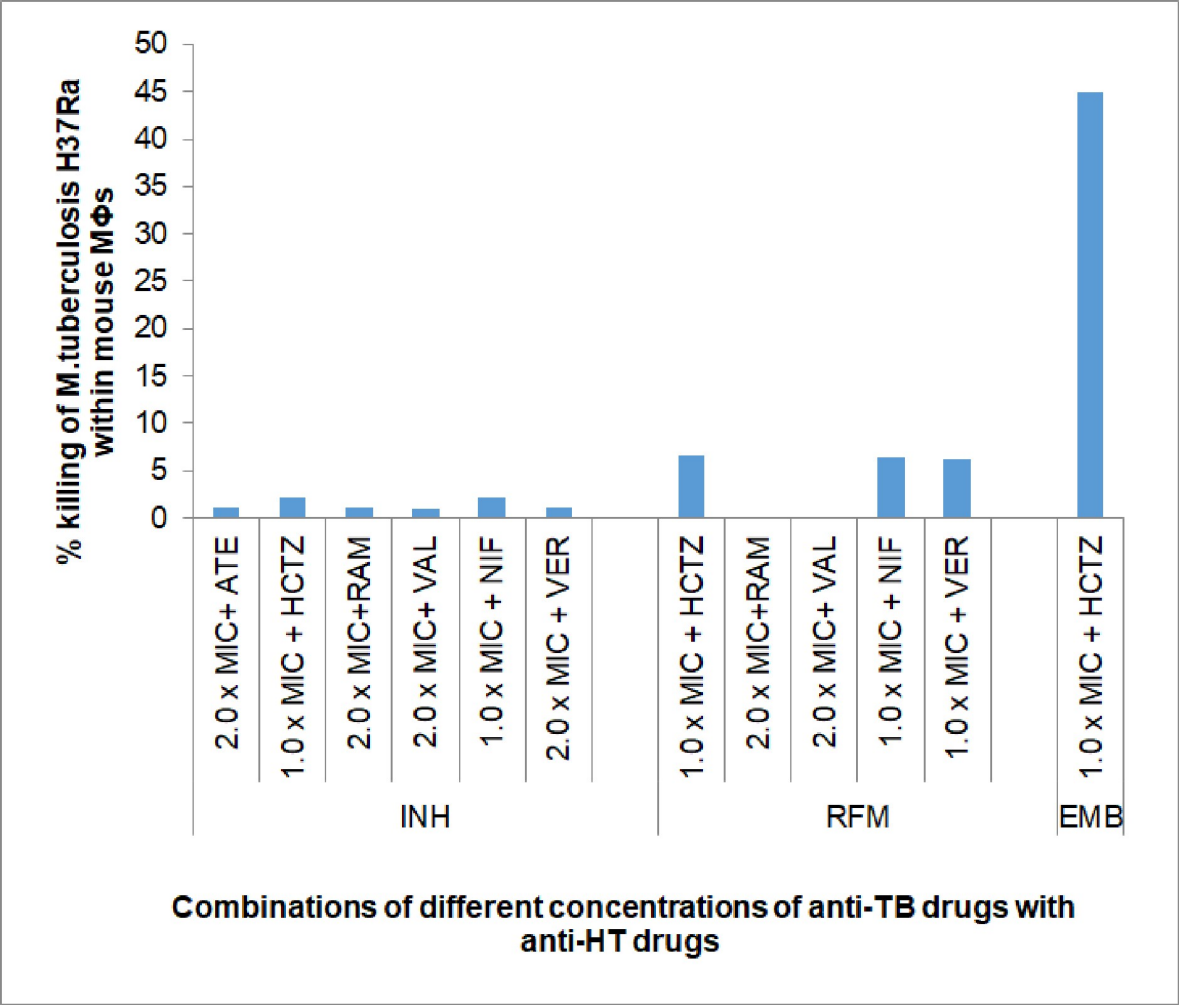
Interactive effect of anti-hypertensive (HT) drugs on the bactericidal activity (% bacterial killing) of anti-TB drugs against intracellular *M*. *tuberculosis* multiplying within mouse bone marrow derived macrophages. Anti-HT drugs: ATE-Atenolol, HCTZ- Hydrochlorothiazide, RAM-Ramipril, VAL-Valsartan, NIF- Nifedipine, VER-Verapamil. Notes: Remaining details are same as in Fig 4.

Depending on the results of inhibitory activity experiments where anti-HT drugs not imposed any effect on the anti-TB drugs, very few combinations were tested to observe the effect of anti-HT drugs on the bactericidal activity of anti-TB drugs. Results [**Table 4 and Fig 5**] showed, that, none of the six anti-HT drugs exerted any effect (termed as ‘None’) on the bactericidal activity of INH. Similarly, five drugs (except ATE) studied in combination with RFM, produced either only very ‘marginally additive’ or ‘no effect’. Only one anti-HT drug, HCTZ enhanced the bacterial killing of EMB by 44.89% with 0.50 x MIC of EMB. Based on the results of *in vitro* experiments, no combination was studied with Streptomycin [**Table 4**].

Together, results [**Tables 3 and 4**] indicate, that, except, Rosiglitazone and Metformin (a key player in the treatment of Type 2 diabetes), all other anti-HG or anti-HT drugs included in the study may be given safely to TB patients receiving treatment under DOTS (Directly Observed Treatment, short course) program, recommended by WHO. Furthermore, synergistic or additive effects obtained with these drug combinations may allow use of reduced doses of drugs. This, in turn may reduce the harmful side effects of these drugs.

## Discussion

In the present study, Oral anti-hyperglycaemic (HG) and anti-hypertensive (HT) drugs, belonging to different, chemical classes and functional mechanisms (**Table 1**) were included. Findings suggest, that, among the seven anti-HG drugs, **Glyburide** and **Repaglinide** were found most promising as they exerted additive effect on the Inhibitory activity and synergistic/ additive effect on the Bactericidal activity of all the four anti-TB drugs. Available literature related to anti-HG drugs indicate that, the drug **Acarbose (ACB)** shown its capability to, disrupt bio-film formation of *Mtb* H37Rv [34] and may, therefore, be a potential candidate towards repurposing for treatment of TB [35]. In the present study, ACB showed ‘additive’ effect on INH and RFM, whereas ‘no effect’ on the activity of EMB and STR to inhibit the growth of *Mtb*. Another drug, **Glyburide**, has been shown to produce potentially adverse side effects on neutrophil and monocyte-mediated immune responses against *Mtb* (reducing IL-1β and IL-8 production) thereby exacerbating susceptibility of diabetes patients to *Mtb* infection [36]. In our study, however, Glyburide has not shown any adverse effect on *in vitro* activity of four anti-TB drugs. It showed ‘additive’ effect with all the 4 drugs. Further, the drug, **Repaglinide** (a K+ATP-channel blocker), has been reported to promote polarization of macrophages towards weakly microbicidal, M2 macrophages, with increased expression of the M2 marker CD206 [37]. This action may increase susceptibility of diabetic individuals to TB and other bacterial infectious disease [38]. In our study, Repaglinide showed an ‘additive effect’ with all the four Anti-TB drugs. Reports on **Metformin**, (used either alone or in combination with other anti-hyperglycaemics) suggest, that, clinically relevant metabolic interactions with metformin, are not possible as this compound is not metabolised and does not inhibit the metabolism of other drugs [38]. Recent data on MET indicate its potential role in improving the effective treatment of TB, suggesting that it can be used as a promising candidate host adjunctive therapy for TB cases [39]. Metformin has also been shown to enhance mycobacterial clearance in mice [40] and is found to lower rates of *M*. *tuberculosis* infection in humans [41]. Other beneficial effects of Metformin reported are, reducing risk of, developing active tuberculosis [42, 43], mortality [44] and relapse [39] and increasing culture conversion [40, 45], thereby supporting successful treatment. All these positive effects, may potentially be associated to glycaemic control, increased production of mitochondrial reactive oxygen species (mROS) and enhanced killing of *M*. *tuberculosis*. Contrary to this, Metformin has been shown to induce anti-inflammatory effects and inhibits pathways such as mammalian target of rapamycin (mTOR) signalling, which are important in the host defence against *Mycobacterium tuberculosis* [46]. These contrary observations of studies may be due to different doses used, heterogeneity of the disease, including cavity or extent of infiltration and bacterial burden. Authors also mentioned that generally anti-TB drugs are effective in treating drug-sensitive TB patients, even with DM. Therefore, additional HDT effect of **Metformin** may not be essential for successful TB treatment. However, it may be a candidate HDT for TB in patients with a high disease burden, such as in that case of cavitary pulmonary TB [45].

In the present study, we found an ‘additive’ effect of **MET** on INH and RFM and no effect on STR. Importantly, FIC values of INH and RFM, were minimum, i.e., 0.125μg/ml, in combination with Metformin. However, the drug (MET) exerted an adverse effect on Ethambutol, a very important first line anti-TB drug, given in the initial Intensive phase of treatment of TB under DOTs program.

Another anti-HG drug, **Rosiglitazone**, improves glycaemic control by improving insulin sensitivity. Being an inducer of CYP2C8, the anti-TB drug Rifampicin has been shown to affect the pharmacokinetics of rosiglitazone in healthy subjects [47]. The study however, provides reasons to believe that co-administration of rifampicin may affect the disposition of rosiglitazone in diabetic patients also, and this may lead to poor glycaemic control. The study, therefore, suggest that, caution should be taken during the co-administration of rifampicin and rosiglitazone [47]. However, in the present study we found that **ROSI** supported the activity of three anti-TB drugs, INH, RFM and STR. Importantly, **ROSI** behaved in a similar manner to that of **MET**, i.e., imparted ‘additive’ effect on INH (FIC 0.125μg/ml) and RFM (FIC 0.25μg/ml), but showed an ‘adverse’ effect on the activity of Ethambutol.

No information is available in the literature regarding any effect of anti-HG drugs, **Acetohexamide** (1st generation-Sulfonylureas) and **Sitagliptin** ((Dipeptidyl peptidase IV inhibitors) on the disease outcome or treatment of tuberculosis. However, the two drugs imparted supportive effect on the activity of all the four anti-TB drugs.

So far, effect of anti-HT drugs is concerned, all showed additive effect on the inhibitory activity of INH and RFM (except **Atenolol**). Drug **Hydrochlorothiazide** also showed ‘additive’ effect on the inhibitory and ‘synergistic’ effect on the bactericidal activity of Ethambutol. Regarding the interaction between anti-hypertensive and anti-TB drugs, available reports showed that the problem of blood pressure control is often observed in TB patients, who are receiving dual, anti- TB and anti-HT treatments. It may be due to the complex drug-drug interactions between the anti-TB drug, rifampicin (an inducer of CYP 450 system) and anti-hypertensive drugs. Therefore, it may be of clinical importance to understand these interactions to help clinicians to suggest safe anti-hypertensive drug(s) for TB patients taking anti-TB medications concomitantly. In this regard, a case report showed that RFM reduced plasma concentration of, anti-hypertensive drugs, **Atenolol** (belong to class of Beta blockers [48], **Nifedipine** (a calcium-channel blocker, CCB- dihydropyridines) and **Verapamil** (CCBs- non-dihydropyridines [49, 50], therefore, reducing their therapeutic efficacy. Recommendations based on case study reviews, suggested that, in patients with hypertension when anti-tuberculosis treatment which include rifampicin is started, the use of calcium channel blockers and classes 1 and 2 β-blockers should be avoided. In such patients, use of, Angiotensin-converting enzyme inhibitors (e.g., **Ramipril**), **Valsartan** (Angiotensin II antagonists) and diuretics (like **hydrochlorothiazide)** should be preferred, as clinically relevant interactions are not expected with these classes of drugs and are shown to be effective [51, 52]. Anti-HT drug, **Verapamil** has been shown to play an important role in the treatment of TB patients in two ways, (i) by transforming non-killing macrophages in to bactericidal, and (ii) improving the therapeutic efficacy of anti-TB drugs by restoring their activity by acting as efflux pump inhibitor. These strategies will be beneficial with the view that they may help in killing of both drug susceptible or drug resistant *Mtb* [53, 54].

Our results showed, that, none of the six anti-HT drugs included in the study, imposed any adverse effect on the inhibitory potential of any of the four Anti-TB drugs. In fact, all the anti-HT drugs except **Atenolol,** showed additive/supportive effect with three prime anti-TB drugs, namely, INH, RFM and EMB with their FICs, <1.00. Effect of **Atenolol** on these drugs was also, ‘None’ (**no effect**). No anti-HT drug exerted any effect on the anti-TB drug Streptomycin.

Many studies [55–58] have shown that combination of drugs provide better treatment outcome for any infectious or life style disease, may be, due to different mechanism of action of different drugs. Also, use of drug combination reduces adverse effects due to use of low concentrations of individual drug(s).

## Conclusion

Co-existence of most prevalent life style disorders like diabetes and/or hypertension increases risk of, failure of anti-TB treatment (identified by positive culture after two months of treatment and relapse after treatment completion), deaths during treatment and development of drug resistant TB. One of the causes of development of drug resistance may be inadequate drug plasma levels due to unrecognized drug-drug interactions.

Potential interferences are expected to occur between anti-TB drugs and other drugs concomitantly administered in TB patients, also suffering with these dual or multi-morbidities. Drug- drug interactions may be produced by drugs/agents that are known to induce or inhibit the cytochrome P450 (CYP) system. Within the limits of present study anti-TB drug RFM is widely known for its effect to induce CYP system, therefore, reducing the serum /plasma concentrations of other co-administered drugs that are CYP substrates and thereby reducing their therapeutic efficacy. However, almost no report is available regarding the effect of anti-HG or anti-HT drugs on the effectiveness of anti-TB drugs.

In our understanding this is the first report demonstrating the effect of anti-HG and/or anti-HT drugs on the inhibitory and bactericidal activities of anti-TB drugs against *M*. *tuberculosis*. Such study is very much required because all these medications, e.g., anti-TB, anti-HG or anti-HT, are to be given together for very long periods. Further, this study is also very important from the view point of the fact that, anti-TB treatment regimens, (i.e., DOTs programme), is based on the set guidelines recommended by WHO and are to be given as such. Clinicians are, therefore, left with the option of selecting anti-hyperglycaemic and/or anti-hypertensive drugs to be given to the TB patients suffering with such co-/multi-morbidities.

## Supporting information

S1 File (DOCX)
