# Supplementary material for: Interactive effect of oral anti-hyperglycaemic or anti–hypertensive drugs on the inhibitory and bactericidal activity of first line anti-TB drugs against M. tuberculosis
Source: PLoS One. 2023 Nov 30;18(11):e0292397. doi: 10.1371/journal.pone.0292397 (PMC10688676; doi:10.1371/journal.pone.0292397)
Supplement: S1 File — (DOCX) [file pone.0292397.s001.docx]

**Supporting Information Data**

**S1aTable.** Original data for the determination of minimum inhibitory concentrations (MICs) of anti-TB drugs, shown in **Tables, 2 and 3,** in the main MS.

|  | Drugs | Test concentrations (µg/ml) | | | | | MIC(µg/ml) |
| --- | --- | --- | --- | --- | --- | --- | --- |
|  | INH | 0.025 | 0.05 | | 0.10 | 0.20 |  |
| Exp 1 |  | + + | + + | | ─ ─ MIC | ─ ─ | 0.10 |
| Exp 2 |  | + + | + + | | ─ ─ MIC | ─ ─ |  |
|  | RFM | 0.10 | 0.20 | 0.40 | | 0.80 |  |
| Exp 1 |  | + + | + + | ─ ─ MIC | | ─ ─ | 0.40 |
| Exp 2 |  | + + | + + | ─ ─ MIC | | ─ ─ |  |
|  | EMB | 2.0 | 4.0 | 8.0 | | 16.0 |  |
| Exp 1 |  | + + | ─ ─ MIC | ─ ─ | | ─ ─ | 4.00 |
| Exp 2 |  | + + | ─ ─ MIC | ─ ─ | | ─ ─ |  |
|  | STR | 1.0 | 2.0 | 4.0 | | 8.0 |  |
| Exp 1 |  | + + | ─ ─MIC | ─ ─ | | ─ ─ | 2.00 |
| Exp 2 |  | + + | ─ ─ MIC | ─ ─ | | ─ ─ |  |
| Exp 1  Exp 2 | No drug  (Control) | + + + +  + + + + | | | | | |

Notes: ─ ─ No bacterial growth.

+ + Bacterial Growth

**S1bTable.** Original data for the determination of MICs of anti-HG and HT drugs against *M. tuberculosis* shown in **Table 2,** in the main MS**.**

| Experiments | Anti-HG drugs | Test concentrations (µg/ml) | | | | MIC(µg/ml) |
| --- | --- | --- | --- | --- | --- | --- |
|  |  | 12.50 | 25.00 | 50.00 | 100.0 |  |
| Exp 1 | Acarbose (ACB) | + + | + + | + + | + + | >100.00 |
| Exp 2 |  | + + | + + | + + | + + |  |
| Exp 1 | Acetohexamide (ACT) | + + | + + | + + | + + | >100.00 |
| Exp 2 |  | + + | + + | + + | + + |  |
| Exp 1 | Glyburide (GLY) | + + | + + | + + | + + | >100.00 |
| Exp 2 |  | + + | + + | + + | + + |  |
| Exp 1 | Metformin (MET) | + + | + + | ─ ─MIC | ─ ─ | 50.00 |
| Exp 2 |  | + + | + + | ─ ─MIC | ─ ─ |  |
| Exp 1 | Repaglinide (REPA) | + + | + + | + + | + + | >100.00 |
| Exp 2 |  | + + | + + | + + | + + |  |
| Exp 1 | Rosiglitazone (ROSI) | + + | + + | ─ ─MIC | ─ ─ | 50 |
| Exp 2 |  | + + | + + | ─ ─MIC | ─ ─ |  |
| Exp 1 | Sitagliptin (SITA) | + + | + + | + + | + + | >100.00 |
| Exp 2 |  | + + | + + | + + | + + |  |
|  | | | | | | |
|  | Anti-HT drugs | Test concentrations (µg/ml) | | | | MIC(µg/ml) |
|  |  | 12.50 | 25.00 | 50.00 | 100.0 |  |
| Exp 1 | Atenolol (ATE) | + + | + + | + + | + + | >100.00 |
| Exp 2 |  | + + | + + | + + | + + |  |
| Exp 1 | Hydrochlorothiazide (HCTZ) | + + | + + | + + | + + | >100.00 |
| Exp 2 |  | + + | + + | + + | + + |  |
| Exp 1 | Ramipril (RAM) | + + | + + | + + | + + | >100.00 |
| Exp 2 |  | + + | + + | + + | + + |  |
| Exp 1 | Valsartan(VAL) | + + | + + | ─ ─MIC | ─ ─ | 50.00 |
| Exp 2 |  | + + | + + | ─ ─MIC | ─ ─ |  |
| Exp 1 | Nifedipine (NIF) | + + | + + | ─ ─MIC | ─ ─ | 50.00 |
| Exp 2 |  | + + | + + | ─ ─MIC | ─ ─ |  |
| Exp 1 | Verapamil (VER) | + + | + + | ─ ─MIC | ─ ─ | 50.00 |
| Exp 2 |  | + + | + + | ─ ─MIC | ─ ─ |  |
|  | No drug  (Control) | + + + ++ | | | | |

Notes: ─ ─ No bacterial growth.

+ + Bacterial Growth

**S2aTable**. Original data for the determination of Fractional Inhibitory Concentrations **(**FICs) of anti-TB drug, Isoniazid (INH) in combination with anti-HG drugs, shown in **Table 3 and Fig 1**, in the main MS.

| Anti-HG Drugs | | | Anti-TB drugs (con. used µg/ml) | | | | | |  |
| --- | --- | --- | --- | --- | --- | --- | --- | --- | --- |
|  | MIC (µg/ml) | Con. Used in Combin-ation  (µg/ml) | 2.0x  MIC  (0.20) | 1.0x  MIC  (0.10) | 0.5x  MIC  (0.05) | 0.25x MIC  (0.025) | 0.125x  MIC  (0.0125) | 0.0625x  MIC  (0.00625) | FIC*  (µg/ml)  Interactive Effect |
| ACB  Exp.1  Exp2 | >100.0 | 100.0 | ─ ─ | ─ ─ | ─ ─ MIC | + + | + + | + + | 0.05/0.1  = 0.50  Additive |
|  |  |  | ─ ─ | ─ ─ | ─ ─ MIC | + + | + + | + + |  |
|  |  |  |  |  |  |  |  |  |  |
| ACT | >100.0 | 100.0 |  |  |  |  |  |  | 0.05/0.1  = 0.50  Additive |
| Exp.1 |  |  | ─ ─ | ─ ─ | ─ ─ MIC | + + | + + | + + |  |
| Exp2 |  |  | ─ ─ | ─ ─ | ─ ─ MIC | + + | + + | + + |  |
|  |  |  |  |  |  |  |  |  |  |
| GLY | >100.0 | 100.0 |  |  |  |  |  |  | 0.0125/0.1  = 0.125  Additive |
| Exp.1 |  |  | ─ ─ | ─ ─ | ─ ─ | ─ ─ | ─ ─ MIC | + + |  |
| Exp2 |  |  | ─ ─ | ─ ─ | ─ ─ | ─ ─ | ─ ─ MIC | + + |  |
|  |  |  |  |  |  |  |  |  |  |
| MET | 50.0 | 25.0 |  |  |  |  |  |  | 0.0125/0.1  = 0.125  Additive |
| Exp.1 |  |  | ─ ─ | ─ ─ | ─ ─ | ─ ─ | ─ ─ MIC | + + |  |
| Exp2 |  |  | ─ ─ | ─ ─ | ─ ─ | ─ ─ | ─ ─ MIC | + + |  |
|  |  |  |  |  |  |  |  |  |  |
| REPA | >100.0 | 100.0 |  |  |  |  |  |  | 0.0125/0.1  = 0.125  Additive |
| Exp.1 |  |  | ─ ─ | ─ ─ | ─ ─ | ─ ─ | ─ ─ MIC | + + |  |
| Exp2 |  |  | ─ ─ | ─ ─ | ─ ─ | ─ ─ | ─ ─ MIC | + + |  |
|  |  |  |  |  |  |  |  |  |  |
| ROSI | 50.0 | 25.0 |  |  |  |  |  |  | 0.0125/0.1  = 0.125  Additive |
| Exp.1 |  |  | ─ ─ | ─ ─ | ─ ─ | ─ ─ | ─ ─ MIC | + + |  |
| Exp2 |  |  | ─ ─ | ─ ─ | ─ ─ | ─ ─ | ─ ─ MIC | + + |  |
|  |  |  |  |  |  |  |  |  |  |
| SITA | >100.0 | 100.0 |  |  |  |  |  |  | 0.05/0.1  = 0.50  Additive |
| Exp.1 |  |  | ─ ─ | ─ ─ | ─ ─ MIC | + + | + + | + + |  |
| Exp2 |  |  | ─ ─ | ─ ─ | ─ ─ MIC | + + | + + | + + |  |

Notes: ─ ─ No bacterial growth.

+ + Bacterial Growth

FIC*= MIC of drug in combination/MIC of drug alone.

Interactive effect- FIC = 1, ‘No effect’

FIC < 1, ‘Additive’ effect

FIC > 1, ‘Adverse’ effect

**S2b Table.** Original data for the determination of Fractional Inhibitory Concentrations **(**FICs) of anti-TB drug, Rifampicin (RFM) in combination with anti-HG drugs. Shown in **Table 3 and Fig 1**, in the main MS.

| Anti-HG Drugs | | | Anti-TB drugs (con. used µg/ml) | | | | | | FIC*  (µg/ml)  Interactive Effect |
| --- | --- | --- | --- | --- | --- | --- | --- | --- | --- |
|  | MIC (µg/ml) | Con. Used in Combin-ation  (µg/ml) | 2.0 x  MIC  (0.80) | 1.0 x  MIC  (0.40) | 0.5 x  MIC  (0.20) | 0.25 x MIC  (0.10) | 0.125 x  MIC  (0.05) | 0.0625x  MIC  (0.025) |  |
| ACB  Exp.1  Exp2 | >100.0 | 100.0 | ─ ─ | ─ ─ | ─ ─ MIC | + + | + + | + + | 0.20/0.40  = 0.50  Additive |
|  |  |  | ─ ─ | ─ ─ | ─ ─ MIC | + + | + + | + + |  |
|  |  |  |  |  |  |  |  |  |  |
| ACT | >100.0 | 100.0 | ─ ─ | ─ ─ | ─ ─ MIC | + + | + + | + + | 0.20/0.40  = 0.50  Additive |
| Exp.1 |  |  |  |  |  |  |  |  |  |
| Exp2 |  |  | ─ ─ | ─ ─ | ─ ─ MIC | + + | + + | + + |  |
|  |  |  |  |  |  |  |  |  |  |
| GLY | >100.0 | 100.0 | ─ ─ | ─ ─ | ─ ─ | ─ ─ MIC | + + | + + | 0.10/0.40  = 0.25  Additive |
| Exp.1 |  |  |  |  |  |  |  |  |  |
| Exp2 |  |  | ─ ─ | ─ ─ | ─ ─ | ─ ─ MIC | + + | + + |  |
|  |  |  |  |  |  |  |  |  |  |
| MET | 50.0 | 25.0 | ─ ─ | ─ ─ | ─ ─ | ─ ─ | ─ ─MIC | + + | 0.05/0.40  = 0.125  Additive |
| Exp.1 |  |  |  |  |  |  |  |  |  |
| Exp2 |  |  | ─ ─ | ─ ─ | ─ ─ | ─ ─ | ─ ─ MIC | + + |  |
|  |  |  |  |  |  |  |  |  |  |
| REPA | >100.0 | 100.0 | ─ ─ | ─ ─ | ─ ─ | ─ ─ MIC | + + | + + | 0.10/0.40  = 0.25  Additive |
| Exp.1 |  |  |  |  |  |  |  |  |  |
| Exp2 |  |  | ─ ─ | ─ ─ | ─ ─ | ─ ─ MIC | + + | + + |  |
|  |  |  |  |  |  |  |  |  |  |
| ROSI | 50.0 | 25.0 | ─ ─ | ─ ─ | ─ ─ | ─ ─ MIC | + + | + + | 0.10/0.40  = 0.25  Additive |
| Exp.1 |  |  |  |  |  |  |  |  |  |
| Exp2 |  |  | ─ ─ | ─ ─ | ─ ─ | ─ ─ MIC | + + | + + |  |
|  |  |  |  |  |  |  |  |  |  |
| SITA | >100.0 | 100.0 | ─ ─ | ─ ─ | ─ ─ | ─ ─ MIC | + + | + + | 0.10/0.40  = 0.25  Additive |
| Exp.1 |  |  |  |  |  |  |  |  |  |
| Exp2 |  |  | ─ ─ | ─ ─ | ─ ─ | ─ ─ MIC | + + | + + |  |

Notes: Remaining details are same as in **S2a Table**.

**S2c Table.** Original data for the determination of Fractional Inhibitory Concentrations **(**FICs) of anti-TB drug, Ethambutol (EMB) in combination with anti-HG drugs. Shown in **Table 3 and Fig 1**, in the main MS.

| Anti-HG- Drugs | | | Anti-TB drugs (con. used µg/ml) | | | | | |  |
| --- | --- | --- | --- | --- | --- | --- | --- | --- | --- |
|  | MIC (µg/ml) | Con. Used in Combin-ation  (µg/ml) | 2.0 x  MIC  (8.0) | 1.0 x  MIC  (4.0) | 0.5 x  MIC  (2.0) | 0.25 x MIC  (1.0) | 0.125 x  MIC  (0.50) | 0.0625 x  MIC  (0.25) | FIC*  (µg/ml)  Interactive Effect |
| ACB  Exp.1  Exp2 | >100.0 | 100.0 | ─ ─ | ─ ─ MIC | + + | + + | + + | + + | 4.0/4.0  =1.0  No effect |
|  |  |  | ─ ─ | ─ ─ MIC | + + | + + | + + | + + |  |
|  |  |  |  |  |  |  |  |  |  |
| ACT | >100.0 | 100.0 | ─ ─ | ─ ─ MIC | + + | + + | + + | + + | 4.0/4.0  =1.0  No effect |
| Exp.1 |  |  |  |  |  |  |  |  |  |
| Exp2 |  |  | ─ ─ | ─ ─ MIC | + + | + + | + + | + + |  |
|  |  |  |  |  |  |  |  |  |  |
| GLY | >100.0 | 100.0 | ─ ─ | ─ ─ | ─ ─ | ─ ─ MIC | + + | + + | 1.0/4.0 =0.25  Additive |
| Exp.1 |  |  |  |  |  |  |  |  |  |
| Exp2 |  |  | ─ ─ | ─ ─ | ─ ─ | ─ ─ MIC | + + | + + |  |
|  |  |  |  |  |  |  |  |  |  |
| MET | 50.0 | 25.0 | + + | + + | + + | + + | + + | + + | > 8.0/4.0  > 2.0  Adverse |
| Exp.1 |  |  |  |  |  |  |  |  |  |
| Exp2 |  |  | + + | + + | + + | + + | + + | + + |  |
|  |  |  |  |  |  |  |  |  |  |
| REPA | >100.0 | 100.0 | ─ ─ | ─ ─ | ─ ─ MIC | + + | + + | + + | 2.0/4.0 =0.50  Additive |
| Exp.1 |  |  |  |  |  |  |  |  |  |
| Exp2 |  |  | ─ ─ | ─ ─ | ─ ─ MIC | + + | + + | + + |  |
|  |  |  |  |  |  |  |  |  |  |
| ROSI | 50.0 | 25.0 | + + | + + | + + | + + | + + | + + | > 8.0/4.0  > 2.0  Adverse |
| Exp.1 |  |  |  |  |  |  |  |  |  |
| Exp2 |  |  | + + | + + | + + | + + | + + | + + |  |
|  |  |  |  |  |  |  |  |  |  |
| SITA | >100.0 | 100.0 | ─ ─ | ─ ─ MIC | + + | + + | + + | + + | 4.0/4.0  = 1.0  No effect |
| Exp.1 |  |  |  |  |  |  |  |  |  |
| Exp2 |  |  | ─ ─ | ─ ─ MIC | + + | + + | + + | + + |  |

Notes: Remaining details are same as in **S2a Table**.

**S2d Table.** Original data for the determination of Fractional Inhibitory Concentrations **(**FICs) of anti-TB drug, Streptomycin (STR) in combination with anti-HG drugs, shown in **Table 3 and Fig 1**, in the main MS.

| Anti-HG- Drugs | | | Anti-TB drugs (con. used µg/ml) | | | | | |  |
| --- | --- | --- | --- | --- | --- | --- | --- | --- | --- |
|  | MIC (µg/ml) | Con. Used in Combin-ation  (µg/ml) | 2.0 x  MIC  (4.0) | 1.0 x  MIC  (2.0) | 0.5 x  MIC  (1.0) | 0.25 x MIC  (0.50) | 0.125 x  MIC  (0.25) | 0.0625 x  MIC  (0.125) | FIC*  (µg/ml)  Interactive Effect |
| ACB  Exp.1  Exp2 | >100.0 | 100.0 | ─ ─ | ─ ─ MIC | + + | + + | + + | + + | 2.0/2.0  = 1.0  No effect |
|  |  |  | ─ ─ | ─ ─ MIC | + + | + + | + + | + + |  |
|  |  |  |  |  |  |  |  |  |  |
| ACT | >100.0 | 100.0 | ─ ─ | ─ ─ MIC | + + | + + | + + | + + | 2.0/2.0  = 1.0  No effect |
| Exp.1 |  |  |  |  |  |  |  |  |  |
| Exp2 |  |  | ─ ─ | ─ ─ MIC | + + | + + | + + | + + |  |
|  |  |  |  |  |  |  |  |  |  |
| GLY | >100.0 | 100.0 | ─ ─ | ─ ─ | ─ ─ | ─ ─ MIC | + + | + + | 0.50/2.0  = 0.25  Additive |
| Exp.1 |  |  |  |  |  |  |  |  |  |
| Exp2 |  |  | ─ ─ | ─ ─ | ─ ─ | ─ ─ MIC | + + | + + |  |
|  |  |  |  |  |  |  |  |  |  |
| MET | 50.0 | 25.0 | ─ ─ | ─ ─ MIC | + + | + + | + + | + + | 2.0/2.0  = 1.0  No effect |
| Exp.1 |  |  |  |  |  |  |  |  |  |
| Exp2 |  |  | ─ ─ | ─ ─ MIC | + + | + + | + + | + + |  |
|  |  |  |  |  |  |  |  |  |  |
| REPA | >100.0 | 100.0 | ─ ─ | ─ ─ | ─ ─ MIC | + + | + + | + + | 1.0/2.0  = 0.50  Additive |
| Exp.1 |  |  |  |  |  |  |  |  |  |
| Exp2 |  |  | ─ ─ | ─ ─ | ─ ─ MIC | + + | + + | + + |  |
|  |  |  |  |  |  |  |  |  |  |
| ROSI | 50.0 | 25.0 | ─ ─ | ─ ─ | ─ ─ MIC | + + | + + | + + | 1.0/2.0  = 0.50  Additive |
| Exp.1 |  |  |  |  |  |  |  |  |  |
| Exp2 |  |  | ─ ─ | ─ ─ | ─ ─ MIC | + + | + + | + + |  |
|  |  |  |  |  |  |  |  |  |  |
| SITA | >100.0 | 100.0 | ─ ─ | ─ ─ | ─ ─ MIC | + + | + + | + + | 1.0/2.0  = 0.50  Additive |
| Exp.1 |  |  |  |  |  |  |  |  |  |
| Exp2 |  |  | ─ ─ | ─ ─ | ─ ─ MIC | + + | + + | + + |  |

Notes: Remaining details are same as in **S2a Table**.

**S3aTable.** Original data for the determination of FICs of anti-TB drug, Isoniazid (INH) with anti-hypertensive (HT) drugs, shown in **Table 3** and **Fig 2**, in the main MS.

| Anti-HG- Drugs | | | Anti-TB drugs (con. used µg/ml) | | | | | |  |
| --- | --- | --- | --- | --- | --- | --- | --- | --- | --- |
|  | MIC (µg/ml) | Con. Used in Combin-ation  (µg/ml) | 2.0 x  MIC  (0.20) | 1.0 x  MIC  (0.10) | 0.5 x  MIC  (0.05) | 0.25 x MIC  (0.025) | 0.125 x  MIC  (0.0125) | 0.0625 x  MIC  (0.00625) | FIC*  (µg/ml)  Interactive Effect |
| ATE  Exp.1  Exp2 | >100.0 | 100.0 | ─ ─ | ─ ─ | ─ ─ MIC | + + | + + | + + | 0.05/0.1  = 0.50  Additive |
|  |  |  | ─ ─ | ─ ─ | ─ ─ MIC | + + | + + | + + |  |
|  |  |  |  |  |  |  |  |  |  |
| HCTZ | >100.0 | 100.0 | ─ ─ | ─ ─ | ─ ─ | ─ ─ MIC | + + | + + | .025/0.10  = 0.25  Additive |
| Exp.1 |  |  |  |  |  |  |  |  |  |
| Exp2 |  |  | ─ ─ | ─ ─ | ─ ─ | ─ ─ MIC | + + | + + |  |
|  |  |  |  |  |  |  |  |  |  |
| RAM | >100.0 | 100.0 | ─ ─ | ─ ─ | ─ ─ MIC | + + | + + | + + | 0.05/0.1  = 0.50  Additive |
| Exp.1 |  |  |  |  |  |  |  |  |  |
| Exp2 |  |  | ─ ─ | ─ ─ | ─ ─ MIC | + + | + + | + + |  |
|  |  |  |  |  |  |  |  |  |  |
| VAL | 50.0 | 25.0 | ─ ─ | ─ ─ | ─ ─ MIC | + + | + + | + + | 0.05/0.1  = 0.50  Additive |
| Exp.1 |  |  |  |  |  |  |  |  |  |
| Exp2 |  |  | ─ ─ | ─ ─ | ─ ─ MIC | + + | + + | + + |  |
|  |  |  |  |  |  |  |  |  |  |
| NIF | >100.0 | 100.0 | ─ ─ | ─ ─ | ─ ─ MIC | + + | + + | + + | 0.05/0.1  = 0.50  Additive |
| Exp.1 |  |  |  |  |  |  |  |  |  |
| Exp2 |  |  | ─ ─ | ─ ─ | ─ ─ MIC | + + | + + | + + |  |
|  |  |  |  |  |  |  |  |  |  |
| VER | 50.0 | 25.0 | ─ ─ | ─ ─ | ─ ─ MIC | + + | + + | + + | 0.05/0.1  = 0.50  Additive |
| Exp.1 |  |  |  |  |  |  |  |  |  |
| Exp2 |  |  | ─ ─ | ─ ─ | ─ ─ MIC | + + | + + | + + |  |

Notes: Remaining details are same as in **S2a Table**.

**S3bTable.** Original data for the determination of FICs of anti-TB drug, Rifampicin (RFM) with anti-hypertensive (HT) drugs, shown in **Table 3** and **Fig 2**, in the main MS.

| Anti-HG- Drugs | | | Anti-TB drugs (con. used µg/ml) | | | | | |  |
| --- | --- | --- | --- | --- | --- | --- | --- | --- | --- |
|  | MIC (µg/ml) | Con. Used in Combin-ation  (µg/ml) | 2.0 x  MIC  (0.80) | 1.0 x  MIC  (0.40) | 0.5 x  MIC  (0.20) | 0.25 x MIC  (0.10) | 0.125 x  MIC  (0.05) | 0.0625 x  MIC  (0.025) | FIC*  (µg/ml)  Interactive Effect |
| ATE  Exp.1  Exp2 | >100.0 | 100.0 | ─ ─ | ─ ─ MIC | + + | + + | + + | + + | 0.40/0.40  =1.0  No effect |
|  |  |  | ─ ─ | ─ ─ MIC | + + | + + | + + | + + |  |
|  |  |  |  |  |  |  |  |  |  |
| HCTZ | >100.0 | 100.0 | ─ ─ | ─ ─ | ─ ─ | ─ ─ MIC | + + | + + | 0.10/0.40  =0.25  Additive |
| Exp.1 |  |  |  |  |  |  |  |  |  |
| Exp2 |  |  | ─ ─ | ─ ─ | ─ ─ | ─ ─ MIC | + + | + + |  |
|  |  |  |  |  |  |  |  |  |  |
| RAM | >100.0 | 100.0 | ─ ─ | ─ ─ | ─ ─ MIC | + + | + + | + + | 0.20/0.40  =0.50  Additive |
| Exp.1 |  |  |  |  |  |  |  |  |  |
| Exp2 |  |  | ─ ─ | ─ ─ | ─ ─ MIC | + + | + + | + + |  |
|  |  |  |  |  |  |  |  |  |  |
| VAL | 50.0 | 25.0 | ─ ─ | ─ ─ | ─ ─ MIC | + + | + + | + + | 0.20/0.40  =0.50  Additive |
| Exp.1 |  |  |  |  |  |  |  |  |  |
| Exp2 |  |  | ─ ─ | ─ ─ | ─ ─ MIC | + + | + + | + + |  |
|  |  |  |  |  |  |  |  |  |  |
| NIF | >100.0 | 100.0 | ─ ─ | ─ ─ | ─ ─ | ─ ─ MIC | + + | + + | 0.10/0.40  =0.25  Additive |
| Exp.1 |  |  |  |  |  |  |  |  |  |
| Exp2 |  |  | ─ ─ | ─ ─ | ─ ─ | ─ ─ MIC | + + | + + |  |
|  |  |  |  |  |  |  |  |  |  |
| VER | 50.0 | 25.0 | ─ ─ | ─ ─ | ─ ─ MIC | + + | + + | + + | 0.20/0.40  =0.50  Additive |
| Exp.1 |  |  |  |  |  |  |  |  |  |
| Exp2 |  |  | ─ ─ | ─ ─ | ─ ─ MIC | + + | + + | + + |  |

Notes: Remaining details are same as in **S2a Table**.

**S3cTable.** Original data for the determination of FICs of anti-TB drug, Ethambutol (EMB) with anti-hypertensive (HT) drugs, shown in **Table 3** and **Fig 2**, in the main MS.

| Anti-HG- Drugs | | | Anti-TB drugs (con. used µg/ml) | | | | | |  |
| --- | --- | --- | --- | --- | --- | --- | --- | --- | --- |
| Expts. | MIC (µg/ml) | Con. Used in Combin-ation  (µg/ml) | 2.0 x  MIC  (8.0) | 1.0 x  MIC  (4.0) | 0.5 x  MIC  (2.0) | 0.25 x MIC  (1.0) | 0.125 x  MIC  (0.5) | 0.0625 x  MIC  (0.25) | FIC*  (µg/ml)  Interactive Effect |
| ATE  Exp.1  Exp2 | >100.0 | 100.0 | ─ ─ | ─ ─ MIC | + + | + + | + + | + + | 4.0/4.0  =1.0  No effect |
|  |  |  | ─ ─ | ─ ─ MIC | + + | + + | + + | + + |  |
|  |  |  |  |  |  |  |  |  |  |
| HCTZ | >100.0 | 100.0 | ─ ─ | ─ ─ | ─ ─ | ─ ─ MIC | + + | + + | 1.0/4.0  =0.25  Additive |
| Exp.1 |  |  |  |  |  |  |  |  |  |
| Exp2 |  |  | ─ ─ | ─ ─ | ─ ─ | ─ ─ MIC | + + | + + |  |
|  |  |  |  |  |  |  |  |  |  |
| RAM | >100.0 | 100.0 | ─ ─ | ─ ─ MIC | + + | + + | + + | + + | 4.0/4.0  =1.0  No effect |
| Exp.1 |  |  |  |  |  |  |  |  |  |
| Exp2 |  |  | ─ ─ | ─ ─ MIC | + + | + + | + + | + + |  |
|  |  |  |  |  |  |  |  |  |  |
| VAL | 50.0 | 25.0 | ─ ─ | ─ ─ MIC | + + | + + | + + | + + | 4.0/4.0  =1.0  No effect |
| Exp.1 |  |  |  |  |  |  |  |  |  |
| Exp2 |  |  | ─ ─ | ─ ─ MIC | + + | + + | + + | + + |  |
|  |  |  |  |  |  |  |  |  |  |
| NIF | >100.0 | 100.0 | ─ ─ | ─ ─ MIC | + + | + + | + + | + + | 4.0/4.0  =1.0  No effect |
| Exp.1 |  |  |  |  |  |  |  |  |  |
| Exp2 |  |  | ─ ─ | ─ ─ MIC | + + | + + | + + | + + |  |
|  |  |  |  |  |  |  |  |  |  |
| VER | 50.0 | 25.0 | ─ ─ | ─ ─ MIC | + + | + + | + + | + + | 4.0/4.0  =1.0  No effect |
| Exp.1 |  |  |  |  |  |  |  |  |  |
| Exp2 |  |  | ─ ─ | ─ ─ MIC | + + | + + | + + | + + |  |

Notes: Remaining details are same as in **S2a Table**.

**S3dTable.** Original data for the determination of FICs of anti-TB drug, Streptomycin (STR) with anti-hypertensive (HT) drugs, shown in **Table 3** and **Fig 2**, in the main MS.

| Anti-HG- Drugs | | | Anti-TB drugs (con. used µg/ml) | | | | | |  |
| --- | --- | --- | --- | --- | --- | --- | --- | --- | --- |
| Expts. | MIC (µg/ml) | Con. Used in Combin-ation  (µg/ml) | 2.0 x  MIC  (4.0) | 1.0 x  MIC  (2.0) | 0.5 x  MIC  (1.0) | 0.25 x MIC  (0.50) | 0.125 x  MIC  (0.25) | 0.0625 x  MIC  (0.125) | FIC*  (µg/ml)  Interactive Effect |
| ATE  Exp.1  Exp2 | >100.0 | 100.0 | ─ ─ | ─ ─ MIC | + + | + + | + + | + + | 2.0/2.0  = 1.0  No effect |
|  |  |  | ─ ─ | ─ ─ MIC | + + | + + | + + | + + |  |
|  |  |  |  |  |  |  |  |  |  |
| HCTZ | >100.0 | 100.0 | ─ ─ | ─ ─ MIC | + + | + + | + + | + + | 2.0/2.0  = 1.0  No effect |
| Exp.1 |  |  |  |  |  |  |  |  |  |
| Exp2 |  |  | ─ ─ | ─ ─ MIC | + + | + + | + + | + + |  |
|  |  |  |  |  |  |  |  |  |  |
| RAM | >100.0 | 100.0 | ─ ─ | ─ ─ MIC | + + | + + | + + | + + | 2.0/2.0  = 1.0  No effect |
| Exp.1 |  |  |  |  |  |  |  |  |  |
| Exp2 |  |  | ─ ─ | ─ ─ MIC | + + | + + | + + | + + |  |
|  |  |  |  |  |  |  |  |  |  |
| VAL | 50.0 | 25.0 | ─ ─ | ─ ─ MIC | + + | + + | + + | + + | 2.0/2.0  = 1.0  No effect |
| Exp.1 |  |  |  |  |  |  |  |  |  |
| Exp2 |  |  | ─ ─ | ─ ─ MIC | + + | + + | + + | + + |  |
|  |  |  |  |  |  |  |  |  |  |
| NIF | >100.0 | 100.0 | ─ ─ | ─ ─ MIC | + + | + + | + + | + + | 2.0/2.0  = 1.0  No effect |
| Exp.1 |  |  |  |  |  |  |  |  |  |
| Exp2 |  |  | ─ ─ | ─ ─ MIC | + + | + + | + + | + + |  |
|  |  |  |  |  |  |  |  |  |  |
| VER | 50.0 | 25.0 | ─ ─ | ─ ─ MIC | + + | + + | + + | + + | 2.0/2.0  = 1.0  No effect |
| Exp.1 |  |  |  |  |  |  |  |  |  |
| Exp2 |  |  | ─ ─ | ─ ─ MIC | + + | + + | + + | + + |  |

Notes: Remaining details are same as in **S2a Table**.

**S4Table.** Original data for the determination of killing of intracellular *M. tuberculosis* (within mouse bone marrow derived macrophages) by anti-TB drugs at different concentrations, e.g., 2.0 x, 1.0 x, 0.50 x and 0.25 x MICs**,** shown in **Table 4** **and Fig 4**, in the main MS.

| Test con. Of drugs | Bacterial killing by anti-TB drugs | | | | | | | |
| --- | --- | --- | --- | --- | --- | --- | --- | --- |
|  | INH | | RFM | | EMB | | STR | |
|  | Bacilli (CFUs/ml) | % killing | Bacilli (CFUs/ml) | % killing | Bacilli (CFUs/ml) | % killing | Bacilli (CFUs/ml) | % killing |
| 2.0 x MIC  Exp. 1  Exp.2  [Average] | 3.40 x 10^3^  1.62 x 10^3^  [2.51 x10^3^] | 98.91 | 00  00  00 | 100.00 | 1.80 x 10^4^  2.20 x 10^4^  [2.00 x 10^4^] | 91.30 | 4.30 x 10^4^  5.36 x 10^4^  [4.83 x 10^4^] | 79.0 |
| 1. x MIC   Exp.1  Exp.2  [ Average] | 3.80 x 10^3^  6.20 x 10^3^  [5.00 x10^3^] | 97.83 | 1.42 x 10  1.58 x 10  [1.50 x 10^4^] | 93.48 | 2.70 x 10^4^  3.30 x 10^4^  [3.00 x 10^4^] | 86.96 | 8.62 x 10^4^  6.30 x 10^4^  [7.36 x 10^4^] | 68.00 |
| 0.50 x MIC  Exp.1  Exp.2  [Average] | 6.32 x 10  7.50 x 10  [6.91 x10^4^] | 70.00 | 6.50 x 10  5.00 x 10  [5.75 x 10^4^] | 75.00 | 1.20 x 10^5^  8.60 x 10^4^  [1.03 x 10^5^] | 55.00 | 1.50 x 10^5^  8.90 x 10^4^  [1.19 x 10^5^] | 48.00 |
| 0.25 x MIC  Exp.1  Exp.2  [Average] | 1.38 x 10^5^  1.52 x 10^5^  [1.45 x 10^5^] | 37.00 | 1.50 x 10  1.26 x 10  [1.38 x 10^5^] | 40.00 | 1.38 x10^5^  1.98 x10^5^  [1.67 x 10^5^] | 27.00 | 1.98 x 10^5^  1.52 x 10^5^  [1.75 x 10^5^] | 24.00 |
| No drug Controls  Day 0 (3H after infection)  Exp.1  Exp. 2  [Average] | | 4.80 x10^4^  6.20 x10^4^  [5.50 x10^4^] | | | | | | |
| Day 5  Exp.1  Exp.2  [Average] | | 2.20 x 10^5^  2.40 x 10^5^  [2.30 x10^5^] | | | | | | |

Notes: 1. Anti-TB drugs- Isoniazid (INH), Rifampicin (RFM), Ethambutol (EMB) and Streptomycin (STR).

1. Colony Forming Units (CFUs)/ml indicate bacterial infection load.

2. Percent (%) killing by the drugs was calculated by considering the bacillary load on Day 5 as 100.0 % bacteria.

3. % bacterial killing was calculated by the average of bacilli (cfu/ml) of the two experiments.

**S5aTable.** Original data for the determination of killing of intracellular *M. tuberculosis* by anti-TB drug, Isoniazid (INH) in combination with anti-HG drugs, shown in **Table 4 and Fig 4,** in the main MS.

| Anti-HG drug(s)  (Con. used in combination,  µg/ml) | Con. of anti-TB drug, x MIC (µg/ml) used in combination | Bacterial killing by anti-TB drug INH | | | % increase in the bacterial killing* | Interactive effect |
| --- | --- | --- | --- | --- | --- | --- |
|  |  | % Killing by Drug  alone# | % killing by drug combination | |  |  |
|  |  |  | Bacilli (CFUs/ml) | % Bacterial  killing |  |  |
| ACB (100.0)  Exp.1  Exp. 2  [Average] | 1.0 x MIC  (0.10) | 97.83 | 00  00  [00] | [100.00] | 2.17 | None |
| ACT(100.0)  Exp.1  Exp. 2  [Average] | 1.0 x MIC  (0.10) | 97.83 | 3.38 x 10^2^  1.68 x 10^2^  [ 2.53 x 10^2^] | [99.89] | 2.06 | None |
| GLY(100.0)  Exp.1  Exp.2  [Average] | 0.25 x MIC  (0.025) | 37.00 | 63.00  75.00  [69.00] | [99.97] | 62.97 | Synergystic |
| MET(50.0)  Exp.1  Exp.2  [Average] | 0.25 x MIC  (0.025) | 37.00 | 00  00  [00] | [100.00] | 63.00 | Synergystic |
| REP(100.0)  Exp.1  Exp.2  [Average] | 0.25 x MIC  (0.025) | 37.00 | 00  00  [00] | [100.00] | 63.00 | Synergystic |
| ROSI(50.0)  Exp.1  EXP.2  [Average] | 0.25 x MIC  (0.025) | 37.00 | 6.92 x 10^2^  5.50 x 10^2^  [ 6.21 x 10^2^] | [99.73] | 62.73 | Synergystic |
| SITA(100.0)  Exp. 1  Exp.2  [Average] | 1.0 x MIC  (0.10) | 97.83 | 25.00  21.00  [23.00] | [99.99] | 2.16 | None |

Notes: 1. Anti-HG drugs used were: ACB- Acarbose, ACT-Acetohexamide, GLY- Glyburide, MET- Metformin, REPA- Repaglinide, ROSI- Rosiglitazone, SITA- Sitagliptin.

2.* % increase in bacterial killing was calculated by subtracting % bacterial killing by the anti-TB drug alone at the respective MIC, from the % bacterial killing by the drug combination(s).

3. # % bacterial killing by the drug alone at the respective MIC is same as shown in S4 Table.

4. Concentrations of anti-TB drugs used in combination were selected on the basis of their inhibitory activity results.

5. Concentration(s) of anti-HG/anti-HT drugs used in combination were 1.0 x MIC. Drugs which showed MIC >100.0 µg/ml were combined at 100.0 µg/ml.

6. Ex vivo experiments for bacterial killing were not performed with the drug combinations which did not show any effect on the inhibitory activity of anti-TB drugs, shown as FIC=1, indicating “No effect”.

7. Criteria for deciding effect of the interaction of anti-HG/ anti-HT drugs on the bactericidal activity of anti-TB drugs was as follows,

(a) ≤ 5% increase in the bacterial killing- showed as `**None**’.

(b) 6-10% increase in the bacterial killing- as, ‘M**arginally additive’.**

(c) 11-20% increase in bacterial killing- as, ‘**Additive’ effect**.

(d) >20% increase in bacterial killing- as, `**Synergistic’ effect**.

**S5bTable.** Original data for the determination of killing of intracellular *M. tuberculosis* by anti-TB drug, Rifampicin (RFM) in combination with anti-HG drugs, shown in **Table 4 and Fig 4,** in the main MS.

| Anti-HG drug(s)  (Con. used in combination,  µg/ml) | Con. of anti-TB drug, x MIC (µg/ml) used in combination | Bacterial killing by anti-TB drug RFM | | | % increase in the bacterial killing* | Interactive effect |
| --- | --- | --- | --- | --- | --- | --- |
|  |  | % Killing by Drug  alone# | % killing by drug combination | |  |  |
|  |  |  | Bacilli (CFUs/ml) | % Bacterial  killing |  |  |
| ACB (100.0)  Exp.1  Exp. 2  [Average] | 1.0 x MIC  (0.40) | 93.48 | 00  00  [00] | [100.00] | 2.17 | None |
| ACT(100.0)  Exp.1  Exp. 2  [Average] | 1.0 x MIC  (0.40) | 93.48 | 3.15 x 10^2^  1.99 x 10^2^  [ 2.57 x 10^2^] | [99.89] | 2.06 | None |
| GLY(100.0)  Exp.1  Exp.2  [Average] | 0.5 x MIC  (0.20) | 75.00 | 00  00  [00] | [100.00] | 62.97 | Synergistic |
| MET(50.0)  Exp.1  Exp.2  [Average] | 0.50 x MIC  (0.20) | 75.00 | 00  00  [00] | [100.00] | 63.00 | Synergistic |
| REP(100.0)  Exp.1  Exp.2  [Average] | 0.50 x MIC  (0.20) | 75.00 | 00  00  [00] | [100.00] | 63.00 | Synergistic |
| ROSI(50.0)  Exp.1  EXP.2  [Average] | 0.50 x MIC  (0.20) | 75.00 | 00  00  [00] | [100.00] | 62.73 | Synergistic |
| SITA(100.0)  Exp. 1  Exp.2  [Average] | 0.50 x MIC  (0.20) | 75.00 | 2.75 x 10^3^  2.27 x 10^3^  [ 2.51 x 10^3^] | [98.91] | 2.16 | None |

Notes: Remaining details are same as in **S5aTable.**

**S5cTable.** Original data for the determination of killing of intracellular *M. tuberculosis* by anti-TB drug, Ethambutol (EMB) in combination with anti-HG drugs, shown in **Table 4 and Fig 4,** in the main MS.

| Anti-HG drug(s)  (Con. used in combination,  µg/ml) | Con. of anti-TB drug, x MIC (µg/ml) used in combination | Bacterial killing by anti-TB drug EMB | | | % increase in the bacterial killing* | Interactive effect |
| --- | --- | --- | --- | --- | --- | --- |
|  |  | % Killing by Drug  alone# | % killing by drug combination | |  |  |
|  |  |  | Bacilli (CFUs/ml) | % Bacterial  killing |  |  |
| ACB (100.0)  Exp.1  Exp. 2  [Average] | Not done |  |  |  |  |  |
| ACT(100.0)  Exp.1  Exp. 2  [Average] | Not done |  |  |  |  |  |
| GLY(100.0)  Exp.1  Exp.2  [Average] | 0.50 x MIC  (2.00) | 55.00 | 37.00  55.00  [46.00] | [99.98] | 44.98 | Synergistic |
| MET(50.0)  Exp.1  Exp.2  [Average] | Not done |  |  |  |  |  |
| REP(100.0)  Exp.1  Exp.2  [Average] | 1.0 x MIC  (4.00) | 86.96 |  | [100.00] | 13.04 | Additive |
| ROSI(50.0)  Exp.1  EXP.2  [Average] | Not done |  |  |  |  |  |
| SITA(100.0)  Exp. 1  Exp.2  [Average] | Not done |  |  |  |  |  |

Notes: Remaining details are same as in **S5aTable.**

**S5dTable.** Original data for the determination of killing of intracellular *M. tuberculosis* by anti-TB drug, Streptomycin (STR) in combination with anti-HG drugs, shown in **Table 4 and Fig 4,** in the main MS.

| Anti-HG drug(s)  (Con. used in combination,  µg/ml) | Con. of anti-TB drug, x MIC (µg/ml) used in combination | Bacterial killing by anti-TB drug STR | | | % increase in the bacterial killing* | Interactive effect |
| --- | --- | --- | --- | --- | --- | --- |
|  |  | % Killing by Drug  alone# | % killing by drug combination | |  |  |
|  |  |  | Bacilli (CFUs/ml) | % Bacterial  killing |  |  |
| ACB (100.0) | Experiment not performed |  |  |  |  |  |
| ACT(100.0) | Experiment not performed |  |  |  |  |  |
| GLY(100.0)  Exp.1  Exp.2  [Average] | 1.00 x MIC  (2.00) | 68.00 | 00  00  [00] | [100.00] | 32.00 | Synergistic |
| MET(50.0) | Experiment not performed |  |  |  |  |  |
| REP(100.0)  Exp.1  Exp.2  [Average] | 1.00 x MIC  (2.00) | 68.00 | 00  00  [00] | [100.00] | 32.00 | Synergistic |
| ROSI(50.0)  Exp.1  EXP.2  [Average] | 1.00 x MIC  (2.00) | 68.00 | 00  00  [00] | [100.00] | 32.00 | Synergistic |
| SITA(100.0)  Exp. 1  Exp.2  [Average] | 1.00 x MIC  (2.00) | 68.00 | 25.00  21.00  [23.00] | [99.73] | 31.73 | Synergistic |

Notes: Remaining details are same as in **S5aTable.**

**S6aTable.** Original data for the determination of killing of intracellular *M. tuberculosis* by anti-TB drug, Isoniazid (INH) in combination with anti-HT drugs, shown in **Table 4 and Fig 5,** in the main MS.

| Anti-HG drug(s)  (Con. used in combination,  µg/ml) | Con. of anti-TB drug, x MIC (µg/ml) used in combination | Bacterial killing by anti-TB drug INH | | | % increase in the bacterial killing* | Interactive effect |
| --- | --- | --- | --- | --- | --- | --- |
|  |  | % Killing by Drug  alone# | % killing by drug combination | |  |  |
|  |  |  | Bacilli (CFUs/ml) | % Bacterial  killing |  |  |
| ATE (100.0)  Exp.1  Exp. 2  [Average] | 2.0 x MIC  (0.20) | 98.91 | 00  00  [00] | [100.00] | 1.09 | None |
| HCTZ (100.0)  Exp.1  Exp. 2  [Average] | 1.0 x MIC  (0.10) | 97.83 | 2.30 x 10^2^  2.76 x 10^2^  [ 2.53 x 10^2^] | [99.89] | 2.06 | None |
| RAM (100.0)  Exp.1  Exp.2  [Average] | 2.00 x MIC  (0.20) | 98.91 | 78.00  60.00  [69.00] | [99.97] | 1.06 | None |
| VAL (50.0)  Exp.1  Exp.2  [Average] | 2.00 x MIC  (0.20) | 98.91 | 4.60 x 10^2^  5.52 x 10^2^  [ 5.06 x 10^2^] | [99.78] | 0.87 | None |
| NIF (50.0)  Exp.1  Exp.2  [Average] | 1.00 x MIC  (0.10) | 97.83 | 00  00  [00] | [100.00] | 2.17 | None |
| VER (50.0)  Exp.1  EXP.2  [Average] | 2.00 x MIC  (0.20) | 98.91 | 00  00  [00] | [100.00] | 1.09 | None |

Notes: 1. Anti-HT drugs used were: ATE-Atenolol, HCTZ- Hydrochlorothiazide, RAM-Ramipril, VAL-Valsartan, NIF- Nifedipine, VER-Verapamil.

2. Remaining details are same as in **S5aTable.**

**S6bTable.** Original data for the determination of killing of intracellular *M. tuberculosis* by anti-TB drug, Rifampicin (RFM) in combination with anti-HT drugs, shown in **Table 4 and Fig 5,** in the main MS.

| Anti-HG drug(s)  (Con. used in combination,  µg/ml) | Con. of anti-TB drug, x MIC (µg/ml) used in combination | Bacterial killing by anti-TB drug RFM | | | % increase in the bacterial killing* | Interactive effect |
| --- | --- | --- | --- | --- | --- | --- |
|  |  | % Killing by Drug  alone# | % killing by drug combination | |  |  |
|  |  |  | Bacilli (CFUs/ml) | % Bacterial  killing |  |  |
| ATE (100.0) | Experiment not performed |  |  |  |  |  |
| HCTZ (100.0)  Exp.1  Exp. 2  [Average] | 1.0 x MIC  (0.40) | 93.48 | 00  00  [00] | [100.00] | 6.52 | Marginally  additive |
| RAM (100.0)  Exp.1  Exp.2  [Average] | 2.00 x MIC  (0.80) | 100.00 | 00  00  [00] | [100.00] | 00 | None |
| VAL (50.0)  Exp.1  Exp.2  [Average] | 2.00 x MIC  (0.80) | 100.00 | 00  00  [00] | [100.00] | 00 | None |
| NIF (50.0)  Exp.1  Exp.2  [Average] | 1.00 x MIC  (0.40) | 93.48 | 2.82 x 10^2^  2.24 x 10^2^  [ 2.53 x 10^2^] | [99.89] | 6.41 | Marginally  additive |
| VER (50.0)  Exp.1  EXP.2  [Average] | 1.00 x MIC  (0.40) | 93.48 | 6.92 x 10^2^  7.80 x 10^2^  [7.36 x 10^2^] | [99.68] | 6.20 | Marginally  Additive |

Notes: Remaining details are same as in **S5a and S6a, Tables.**

**S6cTable.** Original data for the determination of killing of intracellular *M. tuberculosis* by anti-TB drug, Ethambutol (EMB) in combination with anti-HT drugs, shown in **Table 4 and Fig 5,** in the main MS.

| Anti-HG drug(s)  (Con. used in combination,  µg/ml) | Con. of anti-TB drug used in combination  x MIC (µg/ml) | Bacterial killing by anti-TB drug EMB | | | % increase in the bacterial killing* | Interactive effect |
| --- | --- | --- | --- | --- | --- | --- |
|  |  | % Killing by Drug  alone# | % killing by drug combination | |  |  |
|  |  |  | Bacilli (CFUs/ml) | % Bacterial  killing |  |  |
| ATE (100.0) | Experiment not performed |  |  |  |  |  |
| HCTZ (100.0)  Exp.1  Exp. 2  [Average] | 0.50 x MIC  (2.00) | 55.00 | 1.85 x 10^2^  3.21 x 10^2^  [ 2.53 x 10^2^] | [99.89] | 44.89 | Synergistic |
| RAM (100.0) | Experiment not performed |  |  |  |  |  |
| VAL (50.0) | Experiment not performed |  |  |  |  |  |
| NIF (50.0) | Experiment not performed |  |  |  |  |  |
| VER (50.0) | Experiment not performed |  |  |  |  |  |

Notes: Remaining details are same as in **S5a and S6a Tables**.

**S6dTable.** Original data for the determination of killing of intracellular *M. tuberculosis* by anti-TB drug, Streptomycin (STR) in combination with anti-HT drugs, shown in **Table 4 and Fig 5,** in the main MS.

No ex vivo drug combination experiment was performed with Streptomycin.
